# Reduction rate as a quantitative knob for achieving deterministic synthesis of colloidal metal nanocrystals

**DOI:** 10.1039/c7sc02833d

**Published:** 2017-08-16

**Authors:** Tung-Han Yang, Kyle D. Gilroy, Younan Xia

**Affiliations:** a The Wallace H. Coulter Department of Biomedical Engineering , Georgia Institute of Technology and Emory University , Atlanta , Georgia 30332 , USA . Email: younan.xia@bme.gatech.edu; b Department of Materials Science and Engineering , National Tsing Hua University , Hsinchu , 30013 , Taiwan; c School of Chemistry and Biochemistry , Georgia Institute of Technology , Atlanta , Georgia 30332 , USA; d School of Chemical and Biomolecular Engineering , Georgia Institute of Technology , Atlanta , Georgia 30332 , USA

## Abstract

Despite the incredible developments made to the synthesis of colloidal metal nanocrystals, it is still challenging to produce them in a reproducible and predictable manner. This drawback can be attributed to the fact that the protocols continue to be built upon qualitative observations and empirical laws. Because of the vast number of intricately entangled experimental parameters in a synthesis, it is almost impossible to predict and control the outcome by knowingly alternating these parameters. In this *Perspective* article, we discuss the recent efforts in pushing nanocrystal synthesis towards a deterministic process based upon quantitative measurements. In particular, we focus on how the reduction rate of a salt precursor can be used as a quantitative knob for predicting and controlling the outcomes of both nucleation and growth. We begin with a brief introduction to the techniques that have been used to extract the kinetic information of a synthesis and then discuss how the reduction rate is correlated with the defect structure, shape/morphology, and elemental distribution of the resultant nanocrystals. We conclude by highlighting some of the recent advances related to *in situ* probing of nanocrystal synthesis, with an emphasis on the real-time, quantitative aspects with regard to both nucleation and growth.

## Abstract 2

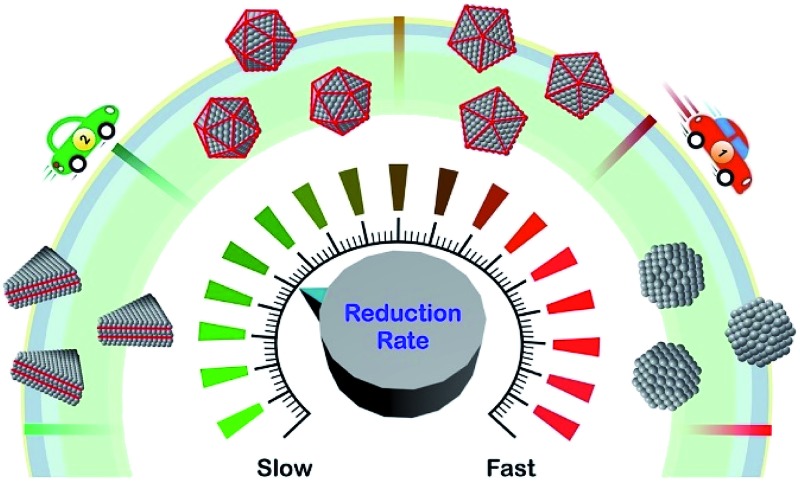
The reduction rate of a salt precursor can be used as a quantitative knob for achieving deterministic synthesis of colloidal metal nanocrystals.

## 1. Introduction

Metal nanocrystals have received ever-increasing interest over the past few decades because of their fascinating performance in various applications, including catalysis, energy conversion and storage, environmental protection, electronics, information storage, photonics, sensing, and biomedicine.^1–9^ Since the physicochemical properties of metal nanocrystals are determined by their size, geometric shape, structure, and composition, most of these applications require the use of nanocrystals with a particular set of physical parameters to optimize their performance towards a specific application. For example, in the case of oxygen reduction reaction, the specific activity (*i.e.*, current density normalized to the electrochemically active surface area) of Pt_3_Ni(111) surface is about 8 times that of the Pt_3_Ni(100) surface and 10 times that of the Pt(111) surface.^10^ In another case, it was found that the activity and selectivity of alkynol hydrogenation strongly correlated with the types of active sites (*e.g.*, plane *vs.* edge atoms) present on the surface of Pd nanocrystals with different shapes.^11^ Such strong structure–function correlations have motivated researchers to develop robust, reproducible, and most importantly, *deterministic* methods for the syntheses of colloidal metal nanocrystals.

**Fig. 1:**
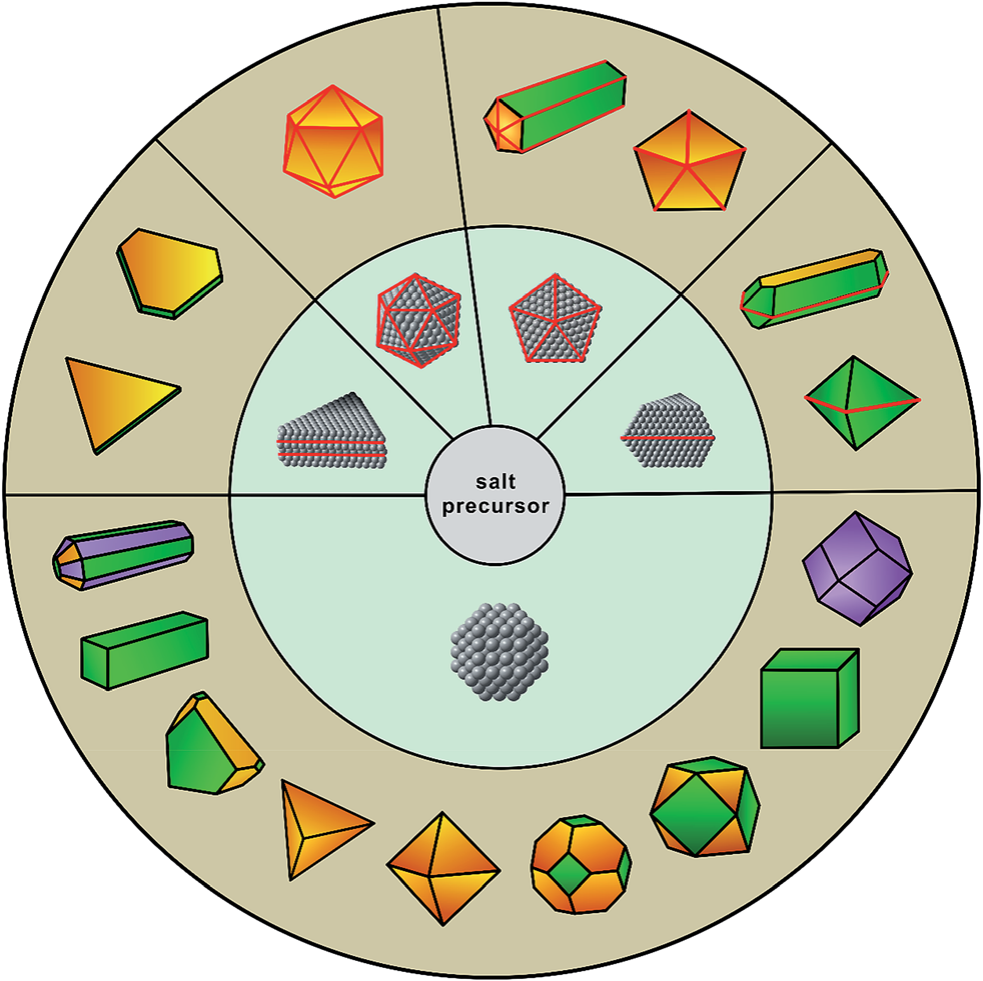
Schematic illustration showing the evolution from salt precursor (in the center), through a homogeneous nucleation process, to five types of seeds (in the middle ring) and then nanocrystals with distinct shapes (in the outer ring). The red lines represent the twin defects or stacking faults in a seed or nanocrystal while the green, purple, and yellow indicate the {100}, {110}, and {111} facets on a nanocrystal, respectively. (Reproduced with permission from ref. 12. Copyright 2017 The Royal Society of Chemistry.)

Thanks to the incredible progress over the last two decades, it is now possible to design and produce metal nanocrystals with a variety of shapes and thus enclosed by the desired facets, as illustrated in Fig. 1.^5,12,13^ Notable examples include 2D nanocrystals such as thin plates or prisms with triangular or hexagonal projections; 1D nanocrystals such as rectangular bars and pentagonal rods or wires; and the more traditional, 0D nanocrystals including cubes, cuboctahedra, octahedra, tetrahedra, rhombic dodecahedra, right bipyramids, decahedra, and icosahedra. In addition, metal nanocrystals enclosed by concave surfaces or high-index facets, including octapods, trisoctahedra, tetrahexahedra, and hexoctahedra, have also been prepared in high yields and with good quality.^14,15^ Traditionally, the synthesis is based upon a one-pot approach where the formation of nanocrystals can be divided into two major steps: (i) homogeneous nucleation – assembly of atoms to generate nuclei and then seeds with a specific internal twin structure once their concentration has reached supersaturation; and (ii) growth – deposition of atoms onto the surfaces of seeds to transform them into nanocrystals with distinctive shapes.^5,6^ Such an approach has been used for hundreds of years, with the first documentation by Michael Faraday for the synthesis of Au colloids by reducing gold chloride with phosphorus (reported in 1856!). In the 1950's, LaMer and co-workers extensively studied the formation of sulfur colloids and developed a qualitative model to describe the nucleation process (*i.e.*, the classical nucleation theory).^16^ In their study, it was found that the nucleation and growth processes were strongly dependent on the concentration of sulfur atoms and could thus be separated into two sequential steps by manipulating the decomposition of a precursor to sulfur. In recent years, with the development of nanochemistry, scientists have identified many non-classical nucleation and growth behaviors in a number of systems, revealing new aspects of crystallization at the nanometer scale.^17^

However, due to the lack of *in situ* characterization tools capable of directly observing reaction pathways at ultra-small time and length scales, it still remains a challenge to unveil the underlying mechanisms associated with a synthesis (the so-called “black box”), which are responsible for the reduction of a salt precursor to generate atoms, followed by their nucleation and then growth into nanocrystals. Nowadays, the far-increased complexity of a protocol often leads to synthesis that is obscured by mysterious mechanisms and plagued by unpredictable results. For example, many groups have reported the synthesis of a variety of Au nanocrystals bound by high-index facets such as {310}, {720}, and {730} by including different amounts of trace Ag^+^ ions in the system.^18–20^ However, the explicit role played by the Ag^+^ ion is still elusive, and many of the proposed theories seem to be contradictory to one another, suggesting that the current models could be misleading or even incorrect. It is not an overstatement that conventional trial-and-error approaches are time-consuming and can actually slow down scientific progression. To remedy this problem, we believe that the nucleation and growth processes, together with the synthetic parameters used to maneuver them, should be derived from mathematical and concrete correlations. To accomplish this goal, we must begin to work towards establishing relationships between key reaction parameters and the resultant physicochemical properties of nanocrystals. In essence, we believe that a synthetic protocol should be founded upon a numerical or *quantitative* basis, which will ultimately lead to deterministic syntheses that are free of randomness, and most importantly, universally applicable among different systems.

Although very little is known about the atomistic details involved in the formation of seeds during a synthesis, several synthetic methods have been developed for experimentally controlling the particular type of seed (*e.g.*, single-crystal, singly twinned, multiply twinned, or stacking fault-lined).^21–24^ It is generally accepted that the internal defect structure of a seed is determined in the earliest stages of a synthesis, which then dictates the shape evolution of a nanocrystal (Fig. 1). For example, single-crystal cubes, octahedra, and tetrahedra all grow from single-crystal seeds; decahedra and icosahedra grow from multiply twinned seeds; bipyramids and beams evolve from singly twinned seeds; and lastly, triangular and hexagonal nanoplates originate from seeds lined with stacking faults. Consequently, one of the most important features involved in the synthesis of nanocrystals is a precise control over the internal defect structure taken by a seed during the nucleation stage. However, it is still a major challenge to approach this issue in the context of one-pot synthesis as the defect structures of seeds and thus the outcomes are determined by a large set of experimental parameters that are intricately entangled. It is also possible to obtain nanocrystals with geometric shapes or symmetries different from that of the seed, but this generally occurs during the growth stage through a physical process commonly referred to as *symmetry breaking* or *symmetry reduction*,^12^ a topic to be discussed in Section 3.2.

**Fig. 2:**
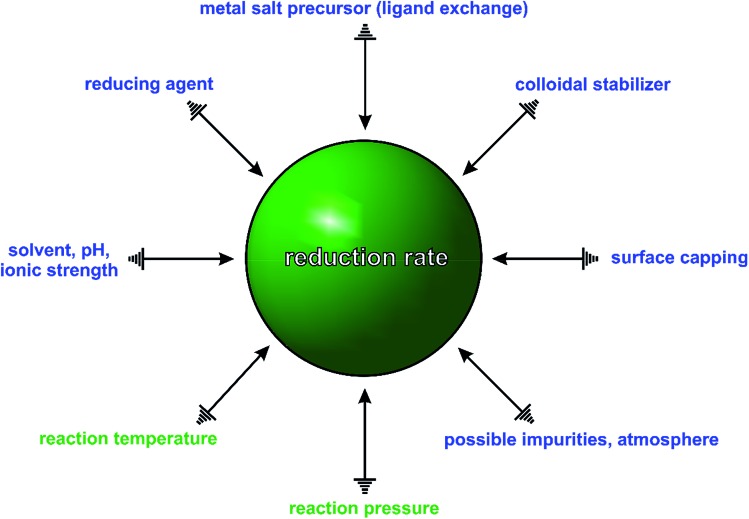
Summary of possible experimental parameters in a typical synthesis of colloidal metal nanocrystals. The physical and chemical parameters are denoted in green and blue, respectively. These parameters can serve as individual knobs to dial the reduction rate, which seems to play the ultimate role in determining the outcome of a synthesis.

In general, a typical synthesis involves a large number of experimental parameters, as illustrated in Fig. 2, including the reaction temperature and pressure; atmosphere and solvent (as well as its pH and ionic strength); type and concentration of salt precursor (ligand exchange), reducing and capping agent, colloidal stabilizer, and possible impurities (known or unknown). Some of these parameters may be prone to changes before or during the synthesis. For example, the salt precursor may change its coordination environment due to ligand exchange, which can have a profound impact on the stability and thus reduction kinetics of the salt precursor. A good example can be found in the ligand exchange between PdCl_4_^2–^ and Br^–^ ions during the synthesis of Pd nanocrystals, resulting in the formation of a series of Pd(ii) complexes (*i.e.*, PdCl_*x*_Br_4–*x*_^2–^, *x* = 0, 1, 2, and 3)^25–27^ with distinctive reduction kinetics.

When any one of the experimental parameters in Fig. 2 is altered, the type and number of seeds, along with the final shapes of nanocrystals will be changed drastically, making it extremely difficult to correlate the products to a particular experimental parameter. For example, in the polyol synthesis of Ag nanocubes, the commercial ethylene glycol is often contaminated with trace amounts of unexpected iron species (*e.g.*, Fe(ii) or Fe(iii) ions) due to the use of steel vessels for both production and storage.^5,28^ These impurities have been shown to induce or influence oxidative etching by coupling with the O_2_ from air and thus the rate of precursor reduction, causing irreproducibility problems. In addition, even the presence of a ppm level of Cl^–^ impurity could also significantly alter the outcome of a synthesis because of its strong impact on oxidative etching.^29^ Taken together, it is not difficult to see why it is challenging to predict and control the outcome of a synthesis of metal nanocrystals. In many cases, only a slight modification to the experimental conditions can drastically impact the outcome.

In recent years, many groups have started to search for a quantitative *knob* that can be adjusted to precisely manipulate the nucleation and growth of nanocrystals in a predictable way. Our group has clearly demonstrated that the reduction rate of salt precursor can serve in such a role.^23,24,30^ As illustrated in Fig. 2, all the experimental parameters can affect the outcome of a synthesis but their roles seems to be executed through the variation of reduction rate. For example, by simply manipulating the initial reduction rate of Pd(ii) precursor in a polyol synthesis, Pd nanocrystals with well-defined defect structures, including single-crystal, multiply twinned, and stacking fault-lined, could be deterministically obtained with high purity, as discussed later in Section 3.1.^23^ In another example, it was shown that the shape and composition of Pd–Pt bimetallic nanocrystals could be easily switched from core–shell octahedra to alloy nanocubes by controlling the ratio between the initial reduction rates of the Pd(ii) and Pt(ii) precursors involved, see Section 3.4.^30^ These examples suggest that the reduction rate of salt precursor not only plays an essential role in determining the outcome of a synthesis but also serves as a quantitative knob for controlling the products of a synthesis.

Regulating the reduction rate extends far beyond simply controlling the internal defect structure of a seed formed in the nucleation stage, because in the subsequent stages it also governs the growth pattern (*e.g.*, symmetric *vs.* asymmetric),^31–34^ growth mode (*e.g.*, island *vs.* layer-by-layer),^35^ and elemental distribution (*e.g.*, core–shell *vs.* alloy).^30^ It should be pointed out that the conclusions derived from most of the early studies are primarily based on *qualitative* observations. As such, the reduction rate has been generally described using qualitative terms such as “slow” and “fast” and the synthesis often involves a trial-and-error approach to reach the optimal synthetic protocol. To guide the optimization of a synthesis more effectively, a quantitative understanding of how reduction kinetics affects nanocrystal formation is of utmost importance. Table 1 summarizes the kinetic parameters, including rate constant (*k*) and activation energy (*E*_a_), which can be obtained by analyzing the kinetics of a given nanocrystal synthesis.^23–25,30,36–42^ As one could imagine, once a systematic database becomes available, it will become possible for almost anybody to design and rationally synthesize metal nanocrystals by selecting a salt precursor with the suitable reduction rate under the proper conditions.

**Table 1:** Summary of quantitative studies of metal nanocrystal syntheses and their corresponding kinetic parameters^*a*^

Precursor	Reductant	Temperature	Additive	Product	Rate constant	Activation energy	Ref.
HAuCl_4_	Formic acid	22 °C	HCl (0.1 M)	Au particles	*k* = 2.26 × 10 min^–1^		36
HAuCl_4_	Polyphenol	22 °C		Au particles	*k* = 4.28 × 10^–3^ min^–1^		37
HAuCl_4_	Photon	22 °C		Au particles	*k*_1_ = 2.04 × 10^–2^ min^–1^		38
					*k*_2_ = 2.39 × 10^1^ min^–1^ M^–1^		
HAuCl_4_ and H_2_PdCl_4_	Sodium citrate	90 °C	CTAB (3.64 mM) and CTAC (10.9 mM)	Au–Pd core–shell icosahedra	*k*_Au_ = 1.68 × 10^–1^ min^–1^		25
					*k*_Pd_ = 1.10 × 10^–2^ min^–1^		
			CTAB (13.0 mM) and CTAC (1.45 mM)	Au–Pd alloyed icosahedra	*k*_Au_ = 3.70 × 10^–2^ min^–1^		
					*k*_Pd_ = 6.00 × 10^–3^ min^–1^		
AgNO_3_	Ethylene glycol	140 °C		Ag particles	*k* = 7.98 × 10^–3^ min^–1^	*E*_a_ = 20.3 kJ mol^–1^	39
Na_2_PdCl_4_	Ethylene glycol	140 °C		Pd truncated octahedra	*k* = 6.72 × 10^–1^ min^–1^	*E*_a_ = 24.0 kJ mol^–1^	23
	Diethylene glycol	140 °C		Pd icosahedra	*k* = 3.34 × 10^–2^ min^–1^	*E*_a_ = 103.2 kJ mol^–1^	
	Triethylene glycol	85 °C		Pd plates	*k* = 1.26 × 10^–4^ min^–1^	*E*_a_ = 110.7 kJ mol^–1^	
Na_2_PdCl_4_	Diethylene glycol	103 °C	Na_2_SO_4_ (0.1 M)	Pd decahedra	*k* = 3.57 × 10^–3^ min^–1^		24
				Pd icosahedra and decahedra	*k* = 3.45 × 10^–4^ min^–1^		
Na_2_PdCl_4_	l-Ascorbic acid	22 °C		Pd concave cubes and particles	*k*_1_ = 6.02 × 10^–2^ min^–1^		40 ^*b*^
					*k*_2_ = 4.20 × 10^–5^ min^–1^ M^–1^		
					*k*′_2_ = 1.94 × 10^–4^ min^–1^ M^–1^		
K_2_PdBr_4_				Pd concave cubes	*k*_1_ = 9.17 × 10^–4^ min^–1^	*E*_a1_ = 131.3 kJ mol^–1^	
					*k*′_2_ = 1.72 × 10^–4^ min^–1^ M^–1^	*E*_a2′_ = 43.4 kJ mol^–1^	
Pd(acac)_2_	Hydrogen gas	22 °C		Pd icosahedra	*k*_1_ = 3.33 × 10^–4^ min^–1^		41
					*k*_2_ = 7.16 × 10^–0^ min^–1^ M^–1^		
Na_2_PdCl_4_ and K_2_PtCl_4_	l-Ascorbic acid	160 °C		Pd–Pt core–shell octahedra	*k*_Pd_ = 1.15 × 10 min^–1^		30
					*k*_Pt_ = 4.27 × 10^–1^ min^–1^		
			KBr (63 mM)	Pd–Pt alloyed nanocubes	*k*_Pd_ = 2.47 × 10^–1^ min^–1^		
					*k*_Pt_ = 3.85 × 10^–1^ min^–1^		
[(C_4_H_9_)_4_N]_5_Na_3_[(1,5-COD)IrP_2_W_15_Nb_3_O_62_]	Hydrogen gas	22 °C		Ir particles	*k*_1_ = 9.33 × 10^–6^ min^–1^		42
					*k*_2_ = 3.56 × 10^1^ min^–1^ M^–1^		

^*a*^The rate constant *k* was derived from the pseudo-first-order kinetic model, while the rate constants *k*_1_ and *k*_2_ correspond to the solution reduction and surface reduction, respectively, which were derived from the Finke–Watzky two-step kinetic model. The activation energy *E*_a_ was obtained using the Arrhenius equation.

^*b*^This reference reported the seed-mediated synthesis of Pd nanocrystals, with the use of Pd cubic seeds. The rate constant *k*′_2_ corresponds to the surface reduction on the Pd cubic seeds. The *E*_a1_ and *E*_a2′_ are the activation energies for solution and surface reduction, respectively.

In this *Perspective* article, we begin by introducing the experimental methods commonly used to quantify the kinetic parameters (*i.e.*, rate constant and activation energy) for the reduction of a salt precursor using measurements based on inductively-coupled plasma mass spectrometry (ICP-MS) or ultraviolet-visible (UV-vis) spectroscopy. We follow this section with discussions on how such measurements can be applied to deepen our understanding of both the nucleation and growth processes and how we can continue to move towards the ultimate goal of achieving a quantitative and deterministic control over nanocrystal synthesis. We then present a series of case studies to highlight how reduction rate can be used as a quantitative knob to precisely and reproducibly tailor the shape of monometallic or bimetallic nanocrystals for a number of systems. In finishing, we highlight and compare new measurement techniques capable of real-time probing the nucleation and growth of nanocrystals, including *in situ* X-ray absorption fine structure (XAFS), high-energy X-ray diffraction (HEXRD), and transmission electron microscopy (TEM). We hope that this *Perspective* article will motivate researchers to take a more progressive action to uncover the explicit role played by reduction kinetics in determining the resultant nanocrystals.

## 2. Quantitative analysis of the reduction kinetics of a salt precursor

### 2.1. Reduction kinetics

In general, the wet-chemical synthesis of metal nanocrystals occurs through the reduction of a salt precursor by a reducing agent. This can be considered a bimolecular elementary reaction due to the nature of collisions and electron transfer occurring between the two species as driven by their difference in reduction potential. As such, the chemical kinetics can be assumed to follow a second-order rate law, with the reduction rate proportional to the concentrations of both reagents. Due to the difficulty in determining the concentration of the reducing agent throughout the course of a synthesis, most studies have concentrated on a pseudo-first-order rate law by supplying the reducing agent in great excess relative to the salt precursor. In this case, the concentration of the reducing agent can be assumed to be constant throughout the course of a synthesis.^23–25,30,36,37,39^ As a result, the reduction rate can be simplified to that of a pseudo-first-order reaction:

1 Rate = *k*′[M^*n*+^][reductant] = *k*[M^*n*+^]

where *k* is the rate constant; and [M^*n*+^] and [reductant] correspond to the concentrations of the salt precursor and reducing agent, respectively. Using this information, the rate constant *k* can be derived by plotting the integrated form of the pseudo-first-order rate law:

2 ln[M^*n*+^]_*t*_ = –*kt* + ln[M^*n*+^]_0_

where *t* is the reaction time; [M^*n*+^]_0_ and [M^*n*+^]_*t*_ represent the concentrations of the salt precursor at the beginning of a synthesis and at a specific time point, respectively.

On the other hand, some research groups including ours have demonstrated that the reduction of a salt precursors can significantly deviate from the pseudo-first-order kinetic model, especially for a synthesis occurring at a relatively slow rate.^38,40–42^ In this case, the reduction rate was typically slow at the initial stage of a synthesis, but increased drastically later on. This unique feature can be modeled using the Finke–Watzky two-step kinetic mechanism that involves pseudo-elementary steps: the precursor is first reduced to zero-valent metal atoms (M^*n*+^ + *n*e^–^ → M^0^; rate constant: *k*_1_), followed by their agglomeration into nuclei (M0*n*) through homogeneous nucleation and then fast autocatalytic surface growth (M^*n*+^ + M0*n* + *n*e^–^ → M0*n*+1; rate constant: *k*_2_) enabled by the just-formed nuclei.^42^ The rate equation can be described using the following equation:

3 Rate = *k*_1_[M^*n*+^] + *k*_2_[M^*n*+^][M0*n*]

where [M0*n*] is the concentration of the nuclei. Under the approximation of [M0*n*] = [M^*n*+^]_0_ – [M^*n*+^], we obtain the following equation for the time-dependent change in precursor concentration, which can be further integrated and expressed in a complex format:4
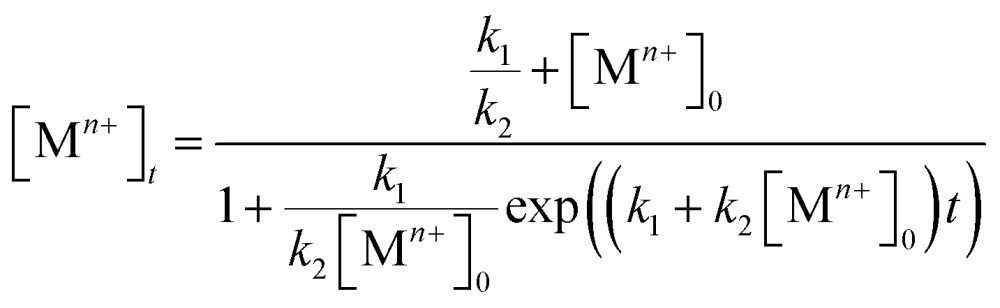



Both the rate constants, *k*_1_ and *k*_2_, for the reduction of a salt precursor can be obtained by fitting the data measured for the time-dependent precursor concentrations to eqn (4). Combined together, it can be concluded that two possible pathways are involved in the nucleation and growth of metal nanocrystals, which can be easily differentiated based on the distinctive features of the time-dependent precursor concentration. When the reduction of precursor follows pseudo-first-order kinetics, the concentration of M^*n*+^ would show exponential decay as a function of reaction time. In comparison, if the reduction of precursor is dominated by the Finke–Watzky two-step kinetics, the time-dependent profile of the M^*n*+^ concentration would show a sigmoidal behavior.

### 2.2. Techniques for quantifying the reduction kinetics

**Fig. 3:**
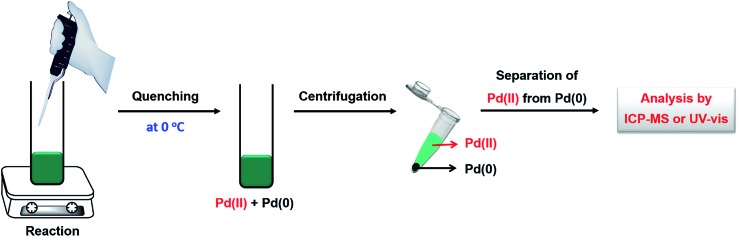
Schematic illustration showing how to quantitatively analyze the reduction kinetics of salt precursor during a synthesis using measurements based on ICP-MS or UV-vis. The reduction of Pd(ii) precursor is simply used as a typical example.

Several analytical techniques, including ICP-MS and UV-vis, have been used to quantitatively measure the reduction kinetics of a precursor in a synthesis of metal nanocrystals.^23,24,30,33,40^ By tracking the concentrations of salt precursor as a function of reaction time using ICP-MS or UV-vis, we are able to derive the rate constant through curve fitting using the equations mentioned in the above section. As shown in Fig. 3, here we use the reduction of a Pd(ii) precursor as a typical example to detail the experimental procedures involved in the measurement of reduction kinetics.^23^ During a synthesis, aliquots are sampled from the reaction solution at different time intervals using a glass pipet, with the first aliquot drawn immediately after the one-shot injection of the Pd(ii) precursor into the reaction solution. The aliquots are immediately cooled to 0 °C to terminate the reduction and thereby preserve the concentration of Pd(ii) ions. It should be pointed out that quickly cooling may not be adequate to completely quench the reaction when a relatively strong reducing agent (*e.g.*, l-ascorbic acid, AA) is involved.^33,40^ To address this issue, the reaction can be more effectively terminated by adding a highly concentrated KBr solution into the sampled solution so that the Pd(ii) precursor can be quickly converted to PdBr_4_^2–^. After quenching, the samples are centrifuged at a high speed of 55 000 rpm for 60 min to precipitate out essentially all the Pd solid products, leaving behind Pd(ii) ions in the supernatant. Otherwise, the supernatant might contain tiny particles (or clusters) that will lead to error in determining the concentration of the remaining precursor. Subsequently, the supernatants are collected and further diluted to a level suitable for ICP-MS or UV-vis measurement. The time-dependent concentrations of Pd(ii) ions remaining in the reaction solution can thus be obtained.

**Fig. 4:**
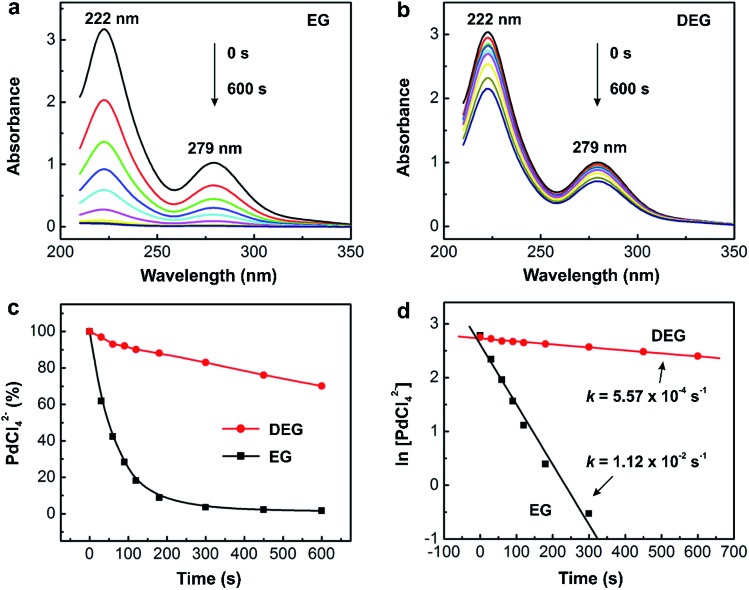
Quantitative analysis of the reduction kinetics of a salt precursor involved in a synthesis as monitored by UV-vis spectroscopy. UV-vis spectra of PdCl_4_^2–^ in (a) ethylene glycol (EG) and (b) diethylene glycol (DEG) reaction solutions at different time points of a reaction. Note that the aliquots are sampled from the reaction solution at different time points and then immediately dissolved in aqueous HCl to prevent the precursor from hydrolysis for the subsequent UV-vis measurement. (c) Plot showing the percentages of PdCl_4_^2–^ remaining in the reaction solutions (calculated using the absorbance at 279 nm) at different time points. (d) Plot showing the pseudo-first-order reduction kinetics involved in the syntheses of single-crystal and multiply twinned Pd nanocrystals in EG and DEG, respectively. (Reproduced with permission from ref. 23. Copyright 2015 American Chemical Society.)

In general, it is feasible to use ICP-MS analysis to measure the concentrations of salt precursor as a function of reaction time for most metal systems due to the high sensitivity and flexibility of ICP-MS. However, the samples prepared for ICP-MS analysis need to be diluted repeatedly with 1% (v/v) aqueous HNO_3_ solution to a level approaching 100 ppb, which is time-consuming and not environmentally friendly. Compared to ICP-MS analysis, UV-vis is much more effective in determining the time-dependent concentrations of a salt precursor. The use of Beer–Lambert's law allows one to exploit the linear dependence between the optical absorbance and the concentration of precursor ions to measure and thus track the percent conversion of the precursor to atoms at different time points. Table 2 summarizes the characteristic absorption peaks of metal–halide complexes commonly used for the syntheses of metal nanocrystals, including AuCl_4_^–^, AuBr_4_^–^, AuI_4_^–^, PdCl_4_^2–^, PdBr_4_^2–^, PdI_4_^2–^, PtCl_4_^2–^, PtBr_4_^2–^ and PtI_4_^2–^. As shown in Fig. 4a and b, the PdCl_4_^2–^ ions dissolved in aqueous HCl exhibited two absorption peaks at 222 and 279 nm that decreased with reaction time. Either one of the peaks could be used to track the concentrations of PdCl_4_^2–^ by comparing to a calibration curve based on standardized PdCl_4_^2–^ solutions.^23^ Fig. 4c shows the as-obtained percentages of PdCl_4_^2–^ remaining in the reaction solution (calculated using the absorbance at 279 nm) as a function of reaction time, which could be further used to derive the rate constant *k* through curve fitting (Fig. 4d).

**Table 2:** Summary of the absorption peak wavelengths for some common salt precursors when they are dissolved in water and ethylene glycol (EG), respectively

Ion	Precursor	Complex	Abs. Peak_water_ (nm)	Abs. Peak_EG_ (nm)
Au(iii)	HAuCl_4_ (0.1 mM) + KCl (100 mM)	AuCl_4_^–^	225	227
	HAuCl_4_ (0.1 mM) + KBr (100 mM)	AuBr_4_^–^	254	259
	HAuCl_4_ (0.1 mM) + KI (100 mM)	AuI_4_^–^	288	293
Pd(ii)	Na_2_PdCl_4_ (0.1 mM) + KCl (100 mM)	PdCl_4_^2–^	279	220
	Na_2_PdCl_4_ (0.1 mM) + KBr (100 mM)	PdBr_4_^2–^	332	286
	Na_2_PdCl_4_ (0.1 mM) + KI (100 mM)	PdI_4_^2–^	408	340
Pt(II)	K_2_PtCl_4_ (0.1 mM) + KCl (100 mM)	PtCl_4_^2–^	215	212
	K_2_PtCl_4_ (0.1 mM) + KBr (100 mM)	PtBr_4_^2–^	^*a*^	^*a*^
	K_2_PtCl_4_ (0.1 mM) + KI (100 mM)	PtI_4_^2–^	331	292

^*a*^The absorption peaks of PtBr_4_^2–^, dissolved in water or ethylene glycol solution, cannot be resolved because the absorption of PtBr_4_^2–^ overlaps with that of KBr species (with strong absorption below 240 nm)^27^ present in the solution. Note that the metal ions (M^*n*+^) should exist in the form of MCl_*m*_^*n*–*m*^, MBr_*m*_^*n*–*m*^, or MI_*m*_^*n*–*m*^ due to the use of an excess amount of Cl^–^, Br^–^, or I^–^ ions in preparing the solution.

Additionally, from the rate constants obtained at different temperatures, the activation energy (*E*_a_) of a reduction reaction involving PdCl_4_^2–^ and a specific polyol can be derived using the Arrhenius equation:

5 *k* = *A*e^–*E*_a_/*RT*^

where *k* is the rate constant; *A* is the frequency factor; *R* is the universal gas constant; and *T* is the reaction temperature. Once the activation energy of a reaction is known, it is straightforward to obtain the rate constants at other temperatures using the Arrhenius equation. This provides a great advantage, especially for reactions that have extremely slow reduction rates so that determination of the rate constant through UV-vis analysis becomes extremely time-consuming.

It should be pointed out that the salt precursor can hydrolyze when it is diluted with water to a relatively low concentration. In this case, the actual species can be identified by analyzing the positions of the major UV-vis absorption peaks. With the use of a proper calibration curve, it is still possible to accurately determine the precursor concentration. For example, it is well documented that the hydrolysis of PdCl_4_^2–^ shifts the absorption peaks due to the formation of hydrated species such as PdCl_*n*_(H_2_O)_4–*n*_^2–*n*^ (*n* < 4).^43–45^ It has been shown that the hydrolysis can be suppressed by using aqueous HCl or KBr solution instead of water for the dilution of PdCl_4_^2–^, enabling the precise determination of precursor concentration from UV-vis analysis.^23,24,30,33,40^ It is worth pointing out that sometimes the absorption peaks of the precursor may overlap with those of other chemical species present in the reaction system such as the reducing agent, colloidal stabilizer, or solvent. This could cause deviations from the actual absorbance of the salt precursor in the reaction solution, as well as the calibration curve derived from the standard solutions (containing the salt precursor only). Therefore, in some cases, it may not be an easy task to precisely determine the precursor concentrations as a function reaction time by relying on UV-vis analysis alone. It is always useful to double check and validate the UV-vis data using ICP-MS measurements.^33^

## 3. Reduction rate as a quantitative knob for controlling nucleation and growth

### 3.1. Correlation between the twin structure of the seed and the initial reduction rate

Since the outcome of a synthesis strongly depends on the twin structure of the seed (Fig. 1), it is highly desirable to precisely control this parameter. Both thermodynamics and kinetics play central roles in determining the formation of seed having a specific twin structure.^6^ When dominated by thermodynamic control, the seed exhibits a global minimum in Gibbs free energy, at which the sum of the surface and volume free energies, twin energy, and strain energy are collectively minimized. The essence of thermodynamic control is to give a system enough thermal energy and an adequately long period of time so that all the atoms in the seed can reach their equilibrium positions to attain the global minimum in Gibbs free energy and thus give the most favorable twin structure at a given size.^46^ However, from the large number of experimental results, the seeds formed in a synthesis could be easily trapped in many thermodynamically less favorable states (*i.e.*, local minima in terms of Gibbs free energy), leading to the formation of kinetic products.^47^ Only recently have syntheses begun to emerge that have a tight control over the internal twin structure of the seed due to the lack of quantitative information about the kinetic parameters.

**Fig. 5:**
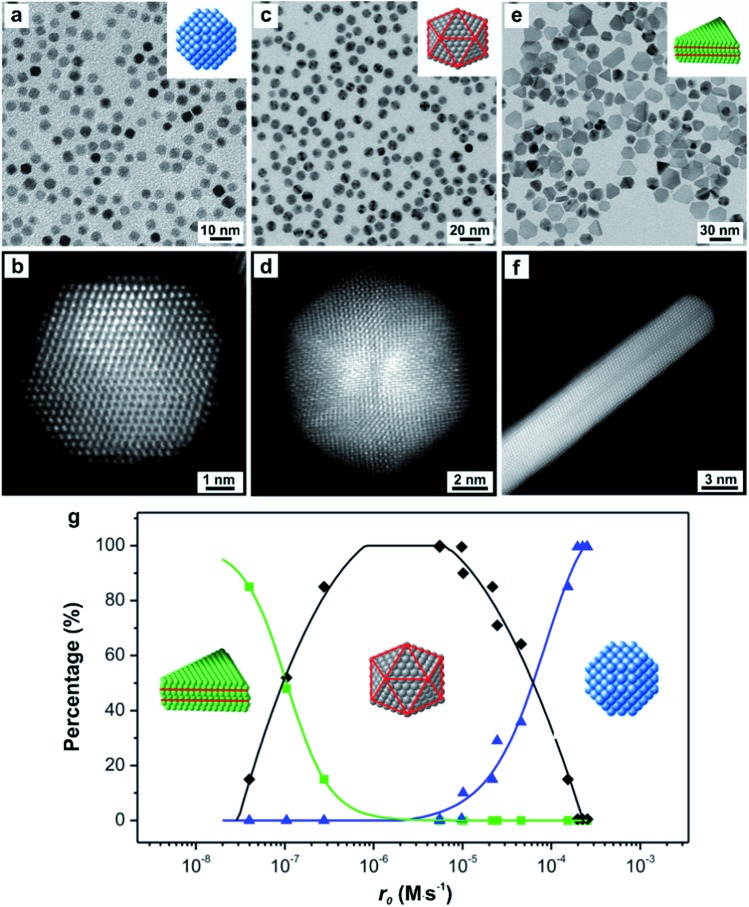
TEM images of (a) truncated octahedra, (c) icosahedra, and (e) nanoplates; with the insets showing the corresponding atomic models of the nanocrystals. Red lines in the atomic models indicate the twin planes or stacking faults. HRTEM images of an individual (b) truncated octahedron (single-crystal), (d) icosahedron (multiply twinned), and (f) nanoplate (stacking fault-lined), revealing the different internal defect structures. Note that the image shown in (f) was taken from the side face of a nanoplate. (g) Percentages of Pd nanocrystals with distinctive twin structures as a function of the initial reaction rate (*r*_0_) of a synthesis, showing single-crystal cuboctahedra (blue curve), multiply twinned icosahedra (black curve), and stacking fault-lined nanoplates (green curve). (Reproduced with permission from ref. 23. Copyright 2015 American Chemical Society.)

As a demonstration, we have developed a quantitative approach through UV-vis measurements to analyze the reduction kinetics of a salt precursor involved in the one-pot synthesis of metal nanocrystals and further reveal the quantitative correlation between the initial reduction rate (*r*_0_) and the internal twin structures of the resultant seed.^23^ Importantly, the syntheses were carried out under nearly identical conditions except for the variation in initial reduction rate, with the overall reaction modeled as pseudo-first-order. Note that the initial reduction rate could be readily obtained by multiplying the rate constant *k* (derived from the pseudo-first-order rate law) by the initial concentration of the precursor. On a quantitative basis, we observed a clear trend where low (*r*_0_ ≈ 10^–8^ M s^–1^), moderate (*r*_0_ ≈ 10^–6^ M s^–1^), and high (*r*_0_ ≈ 10^–4^ M s^–1^) initial reduction rates corresponded to Pd seeds with plate-like (stacking fault-lined), icosahedral (multiply twinned), and truncated octahedral (single-crystal) structures, respectively, as illustrated in Fig. 5. The internal twin structure of the seed in a given synthesis can therefore be predicted based on this quantitative correlation. For a synthesis with a known rate constant *k* derived experimentally, the critical precursor concentration required for the formation of seed with a desired internal twin structure can be readily estimated. For example, when the reaction was conducted in diethylene glycol (DEG) at 130 °C with a relatively high rate constant, stacking-fault-lined nanoplates could still be obtained as long as the concentration of PdCl_4_^2–^ precursor was reduced to 1.76 × 10^–4^ M, corresponding to an initial reduction rate of 5.56 × 10^–8^ M s^–1^.

From an atomic-scale point of view, the difference between single-crystal structures and those containing planar defects is the packing of atoms. For a face-centered cubic (fcc) metal, single-crystal seeds have a uniform cubic close-packed (ccp) lattice, whereas defect-lined seeds have a ccp-lattice with short planar segments featuring a hexagonal close-packed (hcp) lattice. Based on our results, it can be concluded that the Pd atoms derived from the reduction of PdCl_4_^2–^ precursor tend to undergo hcp packing to generate seeds with stacking faults and/or twin planes when the PdCl_4_^2–^ ions are reduced at relatively slow rates. It is worth emphasizing that this new development not only represents a major step towards the quantitative analysis of the reduction kinetics involved in a synthesis but also demonstrates that reduction rate can serve as a quantitative knob for enabling deterministic syntheses of seeds with different types of twin structures.

**Fig. 6:**
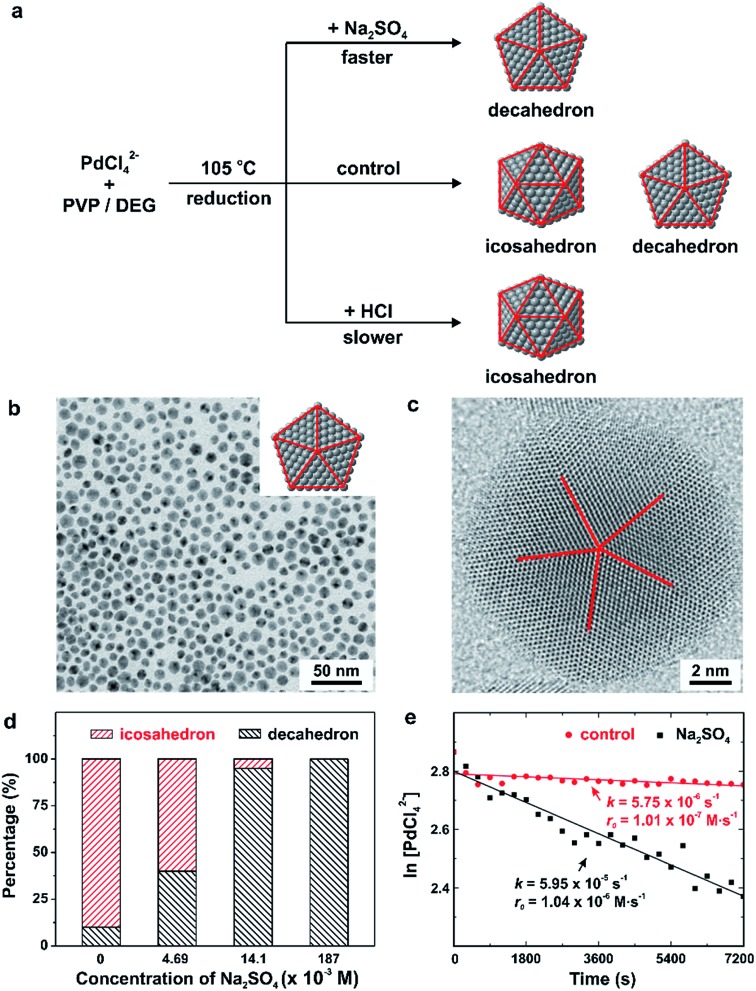
(a) Summary of the different experimental conditions that lead to the formation of Pd decahedra, icosahedra, and a mixture of decahedra and icosahedra in the final products. Note that the twin planes are lined in red. (b) TEM image of Pd decahedra and (c) HAADF-STEM image of an individual particle selected from the sample shown in (b). (d) Percentages of decahedra and icosahedra as a function of the concentration of Na_2_SO_4_ introduced into the synthesis. (Reproduced with permission from ref. 22. Copyright 2014 American Chemical Society.) (e) Plot of ln[PdCl_4_^2–^] as a function of reaction time for the control (sulfate-free) and Na_2_SO_4_^–^ introduced (sulfate-mediated) synthesis, together with the corresponding rate constants and initial reduction rates. (Reproduced with permission from ref. 24. Copyright 2016 American Chemical Society.)

We have also applied the concept of quantitative kinetic control to investigate the synthesis of different types of multiply twinned seeds with a decahedral *vs.* icosahedral shape, as shown in Fig. 6.^22,24^ As revealed by computational simulations^46,48^ and experimental studies^49^ carried out *in vacuo*, multiply twinned structures of Pd are thermodynamically favored only at very small sizes, with the total number of Pd atoms fewer than 309. In light of this constraint, it is not hard to understand that we have to rely on kinetically controlled methods to synthesize Pd decahedra and icosahedral nanocrystals of relatively large sizes. Typically, a synthesis can produce decahedral and icosahedral nanocrystals simultaneously due to the fact that only a slight modification to the reduction rate is needed to favor the formation of one over the other.^22,24,50^ For example, from our early study reported in 2014, a mixture of tiny decahedra (10%) and icosahedra (90%) was obtained when PdCl_4_^2–^ was reduced in diethylene glycol at a reaction temperature of 105 °C, as shown in Fig. 6a and d.^22^ To address this issue, we introduced additives (*e.g.*, Na_2_SO_4_ and HCl) at suitable amounts to stimulate the correct reduction kinetics for the formation decahedral and icosahedral seeds, respectively. The formation of pure decahedra (Fig. 6b and c) prevailed when the reduction rate of PdCl_4_^2–^ was accelerated through the introduction of Na_2_SO_4_, whereas slowing down the reduction through the addition of HCl led to the formation of icosahedral seeds with a purity approaching 100%. From these data described using qualitative terms, we concluded that the reduction rate of PdCl_4_^2–^ followed the trend of decahedron > decahedron + icosahedron > icosahedron.

More recently, to further quantify the effect of Na_2_SO_4_ additives on the polyol synthesis of Pd decahedral nanocrystals, we conducted kinetic studies using UV-vis measurements to derive the rate constants for the Na_2_SO_4_-free (*i.e.*, the control experiment in Fig. 6a) and Na_2_SO_4_-mediated reactions, as shown in Fig. 6e.^24^ The rate constants *k*, derived from the pseudo-first-order rate law, were *k* = 5.75 × 10^–6^ and 5.95 × 10^–5^ s^–1^ for the Na_2_SO_4_-free and Na_2_SO_4_-mediated reactions, respectively. It supports the previously stated hypothesis that Na_2_SO_4_ additives tend to speed up the reduction kinetics.^22^ When the *k* values were multiplied by the initial concentration of PdCl_4_^2–^ (1.76 × 10^–2^ M), we further obtained initial reduction rates of *r*_0_ = 1.01 × 10^–7^ and 1.04 × 10^–6^ M s^–1^ for the Na_2_SO_4_-free and Na_2_SO_4_-mediated reactions, respectively. This quantitative information is in good agreement with the established correlation between the twin structure of the seed and the initial reduction rate in Fig. 5g.^23^ The multiply twinned seeds would appear in the products when the initial reduction rate was controlled in the range of 1.97 × 10^–4^ to 4.0 × 10^–7^ M s^–1^, and the initial reduction rate required for the formation of decahedral seeds was faster than that for icosahedral seeds. These results again confirmed that the reduction rate can be used as a quantitative knob for controlling the internal twin structure of the seed when a one-pot approach is used for the synthesis of metal nanocrystals.^23,24,30^

### 3.2. Symmetry breaking during nanocrystal growth

In a one-pot synthesis, nucleation and growth occur simultaneously. Although these two competing processes can be stimulated under similar experimental conditions, their optimal parameters tend to differ substantially, resulting in the formation of nanocrystals with diverse sizes and shapes. Compared to the one-pot approach, seed-mediated growth, in which a salt precursor is introduced into a reaction solution containing well-defined seeds to facilitate their growth into specific nanocrystals, offers a simple and effective means to separate growth from nucleation. Note that the seeds discussed herein are different from nuclei in that the former have a fixed internal twin structure. For the seed-mediated growth involving one-shot injection of the precursor solution, the numerous atoms generated from the reduction of the precursor within a short period of time (at a fast reduction rate) may still undergo homogeneous nucleation to generate new nuclei instead of growing from the surface of the preformed seeds. This is especially true when the number of possible nucleation sites provided by the seed is not sufficiently large to accommodate all the atoms generated from the precursor reduction.

Alternatively, the use of a syringe pump provides an opportunity to introduce the precursor solution dropwise into a suspension of the preformed seeds and thus eliminate homogeneous nucleation by keeping the concentration of atoms below the threshold value of supersaturation.^9,33^ More significantly, our group previously demonstrated a straightforward method for breaking the cubic or icosahedral symmetry of the seeds by simply manipulating the reduction kinetics of a precursor through the assistance of a syringe pump.^31–34^ For example, we showed that Ag or Au atoms could be selectively deposited on a specific number of faces (ranging from one to six) on a Pd cubic seed to generate asymmetric bimetallic nanocrystals.^31,32^ By tuning the injection rate of the AgNO_3_ or HAuCl_4_ solution using a syringe pump, we could tightly modulate the reduction rate. It is worth pointing out that all these studies were based on a trial-and-error approach when manipulating the experimental parameters (*e.g.*, the injection rate of precursor, temperature, and the type of reducing agent). The situation did not change until 2015 when we developed a model to quantitatively analyze the growth pattern (symmetric *vs.* asymmetric) of Pd cuboctahedral seeds under the dropwise addition of Pd(ii) precursor, as discussed in the following paragraphs.^33^

**Fig. 7:**
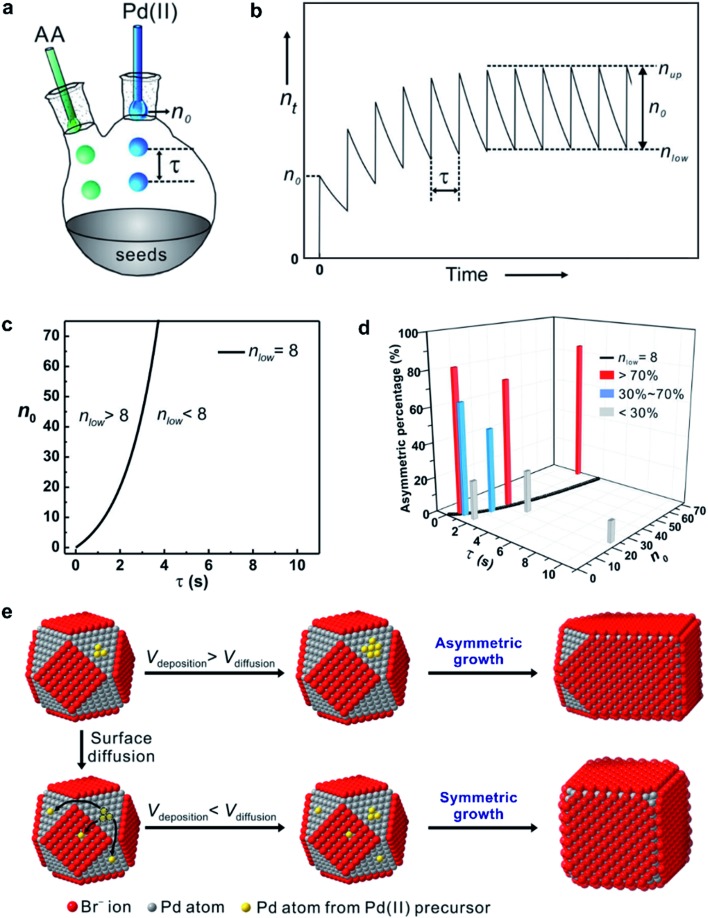
(a) Illustration of the experimental setup used for dropwise addition of Pd(ii) precursor solution into a reaction solution containing Pd cuboctahedral seeds. (b) Plot showing the number (*n*_t_) of Pd(ii) ions in the reaction solution as a function of reaction time. (c) Plot showing *n*_low_ as a function of *n*_0_ and *τ*. The black solid line is *n*_low_ = 8. (d) Plot showing the percentages of asymmetric Pd nanocrystals formed under different reaction conditions by adjusting the variables *n*_0_ and *τ*. (e) Schematic illustrating how the relative ratio of the deposition rate (*V*_deposition_) to the diffusion rate (*V*_diffusion_) affect the growth pattern (symmetric *vs.* asymmetric) when Pd cuboctahedral seeds are used. (Reproduced with permission from ref. 33. Copyright 2015 American Chemical Society.)

Fig. 7a shows the experimental setup used for the seed-mediated growth of Pd nanocrystals, where two aqueous solutions containing a Pd(ii) precursor and ascorbic acid (AA, a reducing agent), respectively, are simultaneously injected at controlled rates from two syringe pumps into a mixture containing Pd cuboctahedral seeds, KBr (a capping agent), and poly(vinyl pyrrolidone) (PVP, a colloidal stabilizer).^33^ To manipulate the reduction kinetics in a quantitative way, two experimental parameters, including the number (*n*_0_) of precursor ions in each drop and the duration of time (*τ*) between adjacent drops, can be controlled by tuning the precursor concentration and injection rate, respectively. In this case, excess AA was supplied relative to the Pd(ii) ions in the synthesis and thus the reduction rate constant *k* could be derived from the ICP-MS data using the pseudo-first-order kinetic model, that is, eqn (1) and (2). From the reduction rate constant *k*, the total number of Pd(ii) precursor ions remaining in the reaction solution at reaction time *t* can be calculated using eqn (6):6
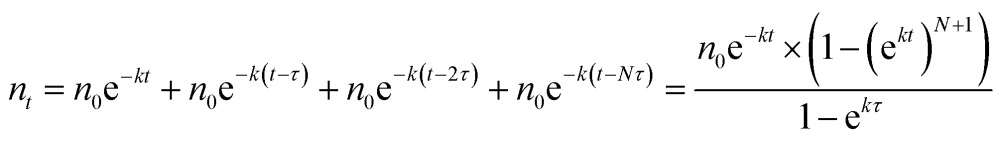
where *n*_*t*_ is the instantaneous number of Pd(ii) precursor in the reaction solution; and *N* represents the total number of Pd(ii)-containing drops injected into the reaction solution from the syringe pump. The plot in Fig. 7b shows the instantaneous number of Pd(ii) ions in the reaction solution as a function of reaction time calculated using eqn (6). It can be clearly seen that the number of Pd(ii) ions quickly reached a steady state after the first few drops have been introduced. In the steady state, the concentration simply oscillates between two limits: the lower limit (*n*_low_) and the upper limit (*n*_up_). The values of *n*_low_ and *n*_up_ can be obtained using eqn (7) and (8):7
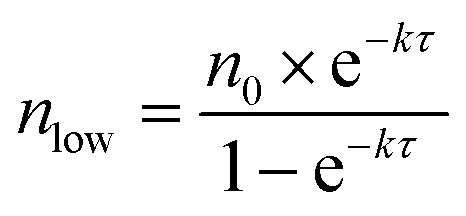

8
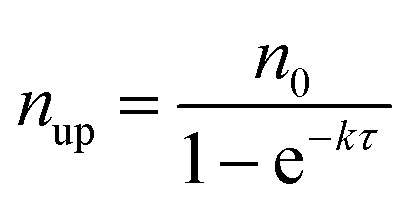



Most importantly, by quantitatively tuning the two experimental variables (*n*_0_ and *τ*) that directly affect the reduction kinetics, we could controllably switch the growth pattern between symmetric and asymmetric. Note that an aspect ratio (length divided by width) of 1.1 was used as the borderline between symmetric and asymmetric growth when analyzing the resultant Pd nanocrystals by TEM imaging. Interestingly, there was a significant transition boundary of around *n*_low_ = 8 that separated the symmetric and asymmetric growth patterns, and this correlation is shown in Fig. 7c and d. When *n*_low_ of the steady state was less than 8 in a given synthesis, the majority of the products were perfect nanocubes with symmetry (*i.e*., *O*_h_) consistent with the cuboctahedral seeds. In comparison, when *n*_low_ was greater than 8, the products were transformed from perfect nanocubes to less symmetric, elongated nanocubes (*i.e.*, nanobars). Fig. 7e schematically illustrates the possible mechanism responsible for the observed correlation between the growth pattern of a cuboctahedral seed and *n*_low_. We argue that the growth pattern of a seed has a strong dependence on the ratio between the atom deposition rate (*V*_deposition_) and the surface diffusion rate (*V*_diffusion_).^33,47^ According to the pseudo-first-order kinetics, the reduction rate and thus *V*_deposition_ are directly proportional to the instantaneous number of precursor ions in the reaction solution. For the first few drops of precursor solution, only a limited number of Pd atoms derived from the reduction of Pd(ii) can be deposited on some of the eight {111} facets of the cuboctahedral seed to generate kinks (activated sites) with a relatively higher surface energy. It is important to note that in this case, the {100} facets are capped by Br^–^ ions and thus protected against Pd deposition. Upon subsequent introduction of precursor solution, the deposition of atoms is largely limited to these activated sites to induce symmetry reduction for the cuboctahedral seed if *V*_deposition_ is greater than *V*_diffusion_ by controlling *n*_low_ above a value of eight. Otherwise, the deposited adatoms can migrate to other sites on the surface of the cuboctahedral seed through surface diffusion, switching the growth pattern from asymmetric to symmetric when *n*_low_ was reduced to a level below 8 by decreasing *n*_0_ and/or extending *τ*. Based upon this quantitative understanding, nanocrystal growth with different degrees of symmetry can be achieved predictably under a specific set of experimental conditions (*e.g.*, precursor concentration and injection rate). Despite the success of this quantitative approach, it should be pointed out that the exposed facets of a seed typically undergo continuous evolution during the growth process, resulting in constant changes to surface energy of a seed in different growth stages. In some cases, it might be necessary to constantly adjust the experimental conditions during the growth process to manipulate the growth pattern or to avoid homogeneous nucleation.

### 3.3. Reduction pathways of a salt precursor

Despite the important role played by the reduction kinetics of a salt precursor in the homogeneous nucleation and seed-mediated growth, as discussed above, the true nature of precursor reduction in a synthesis is still a controversial issue. Ultimately, this is due to the lack of an *in situ* characterization tool capable of deriving necessary quantitative data. Based on the recent development of quantitative methods, we designed a set of kinetic experiments to expand our understanding of the reduction pathway undertaken by a precursor, as discussed in this section.^40^

**Fig. 8:**
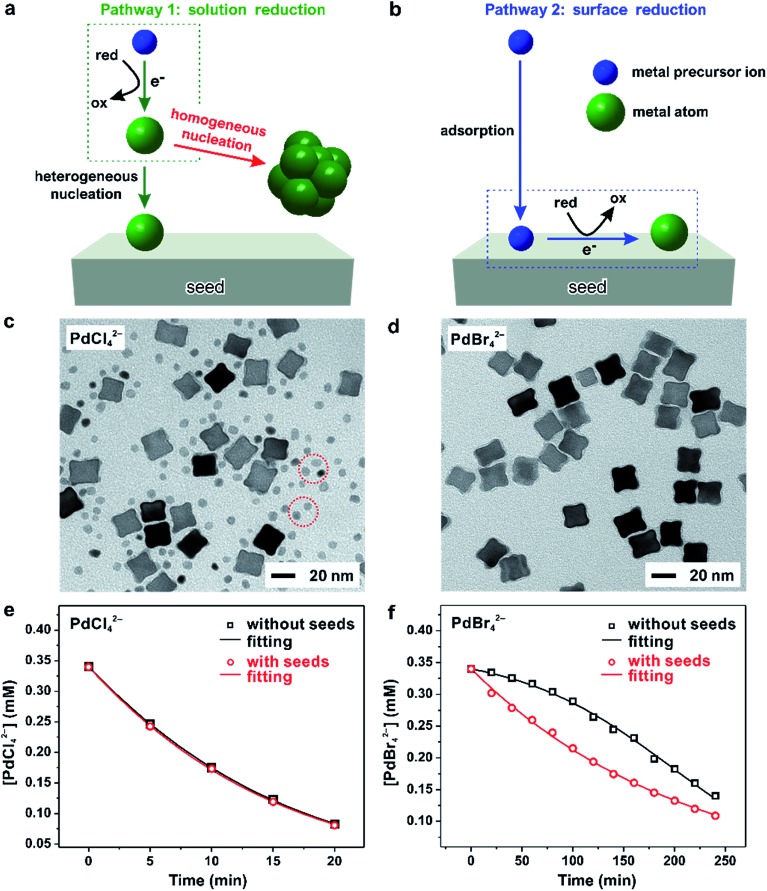
(a) Pathway 1: solution reduction – where the precursor ion is first reduced within the solution, and the release atoms heterogeneously nucleation on the surface of the seed. (b) Pathway 2: surface reduction – where the precursor ion first adsorbs onto the surface of a seed and then gets reduced into atom. TEM images of Pd nanocrystals obtained where (c) PdCl_4_^2–^ and (d) PdBr_4_^2–^ were used as the precursors, respectively, in the presence of 18 nm Pd nanocubes as the seeds. Note that the tiny nanoparticles marked in panel (c) were formed due to homogeneous nucleation. Time-dependent concentrations of (e) PdCl_4_^2–^ and (f) PdBr_4_^2–^ remaining in the reaction solutions as determined using UV-vis in the absence (blue lines) or presence of Pd cubic seeds (red lines). (Reproduced with permission from ref. 40. Copyright 2017 American Chemical Society.)

Fig. 8a and b, illustrates the two possible reduction pathways for a precursor ion in a seed-mediated synthesis.^40^ When a precursor ion is introduced into a solution containing a reducing agent and preformed seeds, it can be reduced in the solution through collision and electron transfer with a reductant molecule (pathway 1: solution reduction), as shown in Fig. 8a. According to the classical LaMer nucleation theory, the concentration of newly formed atoms is then gradually increased as the reaction proceeds, and eventually reaches a critical concentration to trigger homogenous and/or heterogeneous nucleation, with the latter one being more favored due to its lower activation energy barrier.^6,16^ However, the presence of preformed seeds provides an alternative route for reducing the precursor. In addition to being reduced in the solution, the precursor could adsorb onto the surface of a seed, followed by reduction through an autocatalytic process (pathway 2: surface reduction), as shown in Fig. 8b.^42^ It can be clearly seen that these two reduction pathways would result in completely different types of products, with the second pathway capable of excluding the involvement of homogeneous nucleation.

To investigate the role played by the reduction pathway in a seed-mediated growth, we used two Pd(ii) precursors, namely PdCl_4_^2–^ and PdBr_4_^2–^, to take advantage of their large difference in reactivity. In general, the reduction potential of PdCl_4_^2–^/Pd is more positive than that of a Br^–^-containing complex and thus has a faster reduction rate.^33^ The synthesis involved the one-shot injection of aqueous PdX_4_^2–^ (X = Cl^–^ or Br^–^) precursor into an aqueous mixture containing AA, PVP, and Pd cubic seeds whose {100} facets were passivated by the chemisorbed Br^–^ ions. As mentioned previously, the hydrolysis of Pd(ii) precursor might occur during a synthesis when the solvent was water, leading to the formation of a series of hydrated species (*i.e.*, PdX_*n*_(H_2_O)_4–*n*_^2–*n*^, where *n* < 4).^43–45^ For simplicity, in the following discussions, we use the chemical formula of PdCl_4_^2–^ or PdBr_4_^2–^ to denote the specific Pd(ii) precursor added into the reaction system. Fig. 8c and d, shows TEM images of Pd nanocrystals obtained when PdCl_4_^2–^ and PdBr_4_^2–^ were used as the precursors to Pd, respectively, during seed-mediated growth at room temperature. In the case of PdCl_4_^2–^, the product contained Pd nanocrystals with two distinctive sizes and shapes (Fig. 8c). The majority of products exhibited a concave surface, implying that the Pd atoms were not deposited on the entire surface but preferentially at the corners and edges of the cubic seed. The site-selective overgrowth could be attributed to the selective chemisorption of Br^–^ ions on the Pd{100} facets, which blocked the deposition on the side faces of a cubic seed.^51^ Apart from concave cubes, there were also tiny particles with a nearly spherical shape and 3.4 nm in diameter, corresponding to the products of homogeneous nucleation. The presence of both concave cubes and tiny particles in the products indicates the coexistence of homogeneous and heterogeneous nucleation. In contrast, homogeneous nucleation was found to be suppressed when PdBr_4_^2–^ was used as the precursor, resulting in solely concave nanocubes (Fig. 8d). Combined together, considering that the only difference between the two syntheses was the type of precursor, these TEM results suggest that the kinetics dictates the reduction pathway and thus the growth pattern.

To quantitatively understand the effect that reduction kinetics has on the reduction pathways of a precursor, we measured the concentrations of precursor ions remaining in the reaction solution at different time points after the precursor solution had been introduced by recording UV-vis spectra. In parallel, we also conducted a set of control experiments in which preformed seeds were absent from the reaction solution to investigate whether the presence of seeds would affect the reduction kinetics/pathway of the precursor. Fig. 8e shows the concentrations of Pd(ii) ions remaining in the reaction solution in the presence or absence of preformed Pd cubic seeds at different time points. The almost identical data in concentration profiles between the two syntheses indicated that the presence of preformed seeds did not significantly affect the reduction kinetics. In contrast, the time-dependent reduction curves for PdBr_4_^2–^ in the presence and absence of preformed Pd cubic seeds gave two completely distinct curves, as shown in Fig. 8f. In contrast to the sluggish reduction of PdBr_4_^2–^ in the absence of preformed seeds, the reduction rate was significantly accelerated in the presence of preformed seeds.

Furthermore, we identified the elementary reactions involved in the synthesis and estimated their corresponding kinetic parameters. Interestingly, the reduction of PdCl_4_^2–^ or PdBr_4_^2–^ by AA in the absence of preformed seeds could be described using the Finke–Watzky two-step growth model, that is, eqn (3) and (4),^42^ by which Pd(ii) ions were reduced to zero-valent atoms (*i.e.*, solution reduction: 
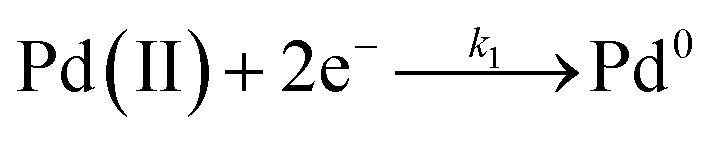
), which then agglomerated to form nuclei (Pd0*n*) through homogeneous nucleation, followed by autocatalytic surface growth (*i.e.*, surface reduction: 
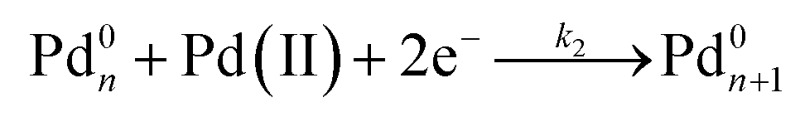
). The rate constants (*k*_1_ and *k*_2_) for the reduction of Pd(ii) precursor in the absence of preformed seeds could thus be extracted from curve-fitting using the Finke–Watzky mechanism. With the introduction of seeds, as defined as Pd0*n*(seed) in the reaction, the seeds would provide extra nucleation sites for the autocatalytic surface growth of Pd(ii) precursor (*i.e.*, surface reduction: 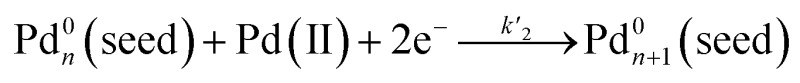). For the case of PdCl_4_^2–^, by constraining the values of *k*_1_ and *k*_2_ obtained from the control experiments (in which the same reduction was carried out in the absence of preformed seeds), the rate constant *k*′_2_ for the surface reduction of PdCl_4_^2–^ by AA on Pd cubic seeds could be obtained. By setting *k*_1_ to its value obtained from the control experiments and *k*_2_ = 0 (because homogeneous nucleation could be excluded, Fig. 8d) for the case of PdBr_4_^2–^, the *k*′_2_ for the surface reduction of PdBr_4_^2–^ by AA on the Pd cubic seeds could be derived. Based on these experimentally determined reduction rate constants, the percentage of solution and surface reduction of the precursor as a function of reaction time could be further calculated by integrating the reduction rates over time. We found that the percentage of solution reduction of PdCl_4_^2–^ reached up to 89% even in the presence of preformed seeds, suggesting that PdCl_4_^2–^ ions were likely reduced to atoms in solution prior to their deposition on the surface of seeds. In comparison, the percentage of surface reduction accounted for over 80% of the PdBr_4_^2–^ reduction in the presence of preformed seeds, implying that the reduction of PdBr_4_^2–^ mainly followed the autocatalytic surface growth pathway where the precursor was reduced on the surface of the seeds rather than in the solution phase, and therefore homogeneous nucleation was completely suppressed.

We also conducted a set of experiments to demonstrate how the reduction mechanism of PdBr_4_^2–^ could be switched by manipulating the reaction temperature and thus the reduction kinetics.^40^ Interestingly, we observed a significant transition from surface reduction to solution reduction when the reaction temperature was switched from low to high for PdBr_4_^2–^ in the presence of seeds. From these quantitative results, we believe that the precursor was reduced on the surface of the seeds through autocatalytic surface growth under slow reaction kinetics whereas it was reduced in the reaction solution at faster reaction rates. Moreover, based on the Arrhenius equation, the activation energies for the solution and surface reduction of PdBr_4_^2–^ were determined to be 131.3 and 43.4 kJ mol^–1^, respectively. In general, the precursor should follow the surface reduction pathway (*i.e.*, autocatalytic surface growth) with a small activation energy of 43.4 kJ mol^–1^. In parallel, before the precursor could possibly reach the surface of a seed, it could also be reduced in the solution phase, but with a much higher activation energy of 131.3 kJ mol^–1^. The solution reduction will be more favorable than surface reduction as long as the reaction temperature is high enough to overcome the activation energy barrier since the precursor ions colloid more frequently with the reductants than with the seeds. These results again demonstrate that the pathway has a strong correlation with the reduction kinetics involved. It should be pointed out that the activation energy barrier to initiate the autocatalytic surface growth is expected to be determined by the types of exposed facets and/or surface sites and/or the presence of twin defects on the seeds because of different catalytic properties of surface structures, which will govern the evolution of seeds into specific nanocrystals through the catalytic reduction route. As a result, it is urgently needed to achieve a systematic, quantitative understanding of the autocatalytic process involved in the seed-mediated growth of metal nanocrystals in the near future.

### 3.4. Extending the quantitative analysis to bimetallic nanocrystals

We and others have also explored the concept of “quantitative knob” to control the relative reduction rates of two salt precursors in an effort to manipulate, more significantly, predict the shape and elemental distribution of bimetallic nanocrystals derived from one-pot syntheses.^30^ In general, bimetallic nanocrystals comprised of two elements can take two distinctive types of structures: core–shell or alloy. As typically found in literature, these two bimetallic structures can be synthesized by co-reducing two salt precursors in one-pot approach.^25,30,52,53^ Skrabalak and co-workers reported the synthesis of Pd–Pt core–shell dendritic bundles and Pd–Pt alloy nanodendrites by co-reducing Pd(acac)_2_ and three Pt(ii) precursors with different metal–ligand environments in oleylamine.^52^ Yang and co-workers demonstrated the synthesis of Au_3_Cu, AuCu, and AuCu_3_ alloy nanocrystals by varying the precursor ratio of Au(CH_3_COO)_3_ to Cu(CH_3_COO)_2_ in a co-reduction process.^53^ Kuo and co-workers selectively obtained Au–Pd alloy and core–shell icosahedral nanocrystals by tuning the ratio of cetyltrimethylammonium chloride (CTAC) to cetyltrimethylammonium bromide (CTAB) in an effort to modulate the reduction kinetics of AuCl_4_^–^ and PdCl_4_^2–^ precursors.^25^ Despite these achievements, there is still much work to be done in terms of precisely manipulating the structures of bimetallic nanocrystals in a predictable way under specific experimental parameters instead of utilizing the more traditional methods based on a trial-and-error approach that is extremely time-consuming, especially in the synthesis of bimetallic nanocrystals.

**Fig. 9:**
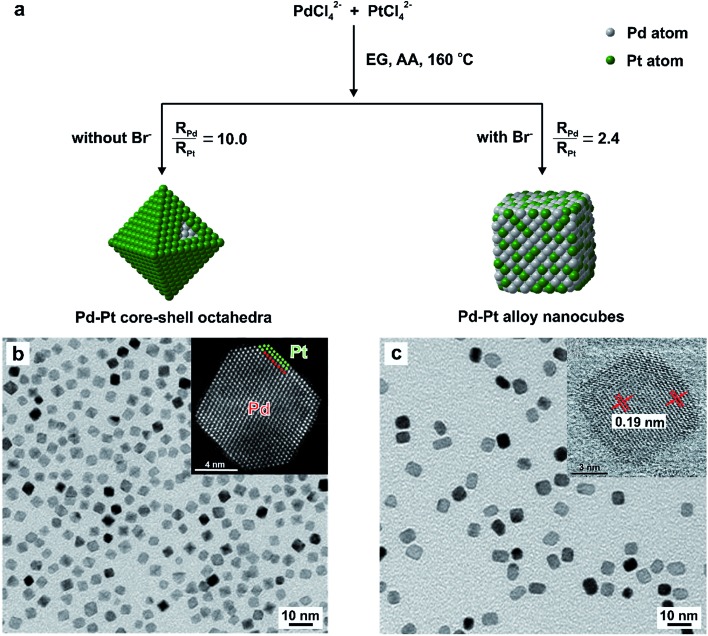
Quantitative analysis of the reduction kinetics responsible for the one-pot synthesis of Pd–Pt bimetallic nanocrystals with different shapes and elemental distribution. (a) Schematic illustration showing the formation of Pd–Pt core–shell octahedra and Pd–Pt alloy nanocubes in the absence and presence of Br^–^ ions, respectively. TEM images of (b) Pd–Pt core–shell octahedra and (c) Pd–Pt alloy nanocubes, respectively. The insets in (b, c) show HAADF-STEM images of individual particles derived from the respective samples. (Reproduced with permission from ref. 30. Copyright 2017 American Chemical Society.)

With Pd–Pt bimetallic nanocrystals as a typical example, we showed that their structures (core–shell *vs.* alloy) can be deterministically obtained by knowing the reduction rates of the Pd(ii) and Pt(ii) precursors, as shown in Fig. 9a.^30^ Specifically, we introduced KBr into the reaction solution to manipulate the reduction rates of these two precursors by influencing their redox potentials *via* fast ligand exchange between Br^–^ and Cl^–^ in PdCl_4_^2–^ or PtCl_4_^2–^. On the basis of reduction rate constants of the precursors derived from the pseudo-first-order kinetic model using ICP-MS analysis, it was demonstrated that Br^–^ ions introduced into the reaction solution played a critical role in altering the initial reduction rate of the Pd(ii) and Pt(ii) precursors and thus the formation of Pd–Pt core–shell octahedra or Pd–Pt alloy nanocubes, as shown in Fig. 9b and c. In the absence of KBr, the ratio between the initial reduction rate of PdCl_4_^2–^ and PtCl_4_^2–^ precursors was approximately 10.0, resulting in the formation of Pd@Pt core–shell octahedral nanocrystals because of the large difference in the initial reduction rates of PdCl_4_^2–^ and PtCl_4_^2–^. From the high-angle annular dark-field scanning-TEM (HAADF-STEM) image shown in Fig. 9b, there was a stark contrast between the Pt shell (brighter) and the Pd core due to the difference in atomic number between these two elements and the number of Pt atomic layers was estimated to be two. When the synthesis was conducted in the presence of Br^–^ ions (*ca.* 63 mM), Pd–Pt alloyed nanocubes enclosed by {100} facets were obtained because the initial reduction rates of the two precursors became comparable to each other, with a ratio of 2.4, because of ligand exchange and thus the formation of PdBr_4_^2–^ and PtBr_4_^2–^. In this case, the alloyed structure of Pd–Pt took a cubic shape owing to the strong capping effect of Br^–^ ions towards the {100} facets.^51^ As further confirmed by a series of quantitative experiments, we concluded that the structure of Pd–Pt bimetallic nanocrystals can be switched from core–shell to alloy when the initial reduction rate of the Pd(ii) precursor is approximately 4–5 times lower than that of the Pt(ii) precursor. This successful demonstration suggests that the initial reduction rates of salt precursors can serve as a quantitative knob for the synthesis bi- and even multi-metallic nanocrystals with desired shapes and compositions for a variety of applications.

## 4. Techniques for probing nucleation and growth *in situ*

As discussed in the above sections, the reaction kinetics involved in the nucleation and growth of metal nanocrystals can be quantitatively analyzed through ICP-MS or UV-vis measurements for tracking the time-dependent precursor concentrations throughout the course of a synthesis. In recent years, a number of *in situ* characterization techniques, including XAFS, HEXRD, and TEM, have started to be explored. These techniques are capable of monitoring the real-time kinetics of a nanocrystal synthesis, without having to worry about possible changes to the samples that might occur during sampling and analysis. In addition, *in situ* techniques with distinctive capabilities can also be used to reveal the other side of a mechanism behind the synthesis.

### 4.1. X-ray absorption fine structure

In one example, Wei and co-workers used *in situ* XAFS to monitor the reduction of PtCl_4_^2–^ precursor and nanocrystal evolution by recording and analyzing the time profiles of Pt–Pt or Pt–Cl bond distances.^54^ They found that when the PtCl_4_^2–^ precursor was reduced with a weak reductant, the intermediate [Cl_3_Pt–PtCl_3_]^4–^ dimer cluster and subsequent aggregates of larger linear Pt_*n*_Cl_*x*_ complexes were formed, which then resulted in the formation of Pt nanowires. In contrast, when a relatively strong reducing agent was introduced, the PtCl_4_^2–^ precursor was fully reduced to zero-valent Pt^0^ atoms that aggregated to generate Pt02, Pt03, and larger Pt0*n* clusters, eventually leading to the formation of Pt nanospheres. Similar kinetics was also observed for the reduction of AuCl_4_^–^ as measured by *in situ* XAFS.^55^ Combined together, the *in situ* XAFS technique is capable of identifying the temporal intermediates formed in the reaction solution, which can satisfy the urgent need for determining the evolving precursor- and nanocrystal-states during the different stages of a synthesis. However, it should be pointed out that the *in situ* XAFS technique may be inadequate in capturing all the aspects of a synthesis due to the fact that this technique is only capable of surveying a small portion of the structural details occurring during a synthesis. When bimetallic clusters are concerned, as previously investigated for the Au–Cu system,^56^ it may be necessary to carry out a series of additional analyses using techniques that include nuclear magnetic resonance (NMR), X-ray photoelectron spectroscopy (XPS), and matrix-assisted laser desorption ionization-time-of-flight-mass spectrometry (MALDI-TOF-MS) to fully characterize all aspects of the clusters.

### 4.2. High-energy X-ray diffraction

**Fig. 10:**
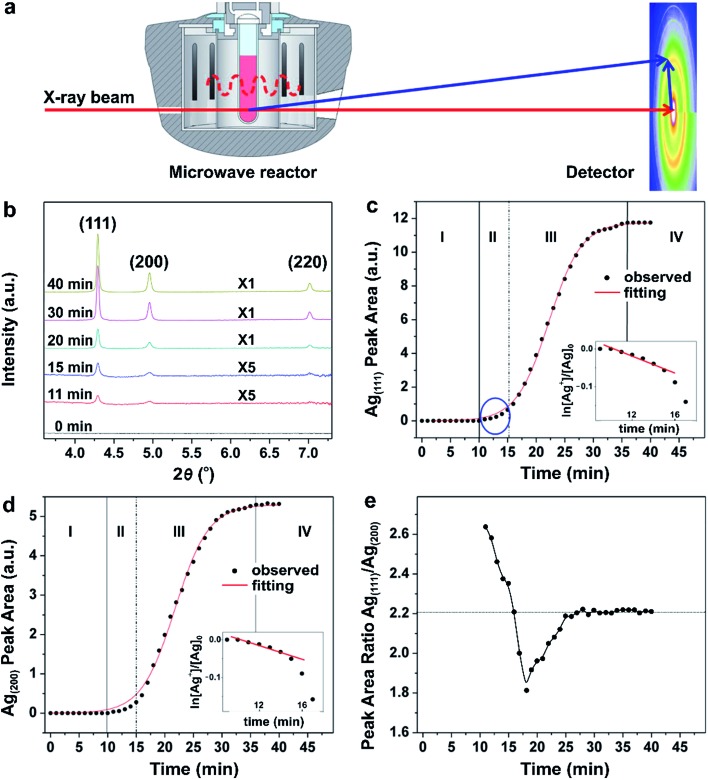
(a) Schematic illustration of the experimental setup used for *in situ* synchrotron HEXRD with a microwave reactor. (b) HEXRD patterns recorded from the microwave synthesis of Ag nanocrystals at different reaction time points. Integrated peak areas of the (c) Ag(111) and (d) Ag(200) at different reaction time points. The red curves in panels (b) and (c) show the best fitting with sigmoidal functions. The four well-defined growth stages (I, II, III, and IV) showed specific characteristics. The plot inserts show the linear fitting of ln([Ag^+^]/[Ag^+^]_0_) as a function of reaction time in stage II, suggesting that the growth follows pseudo-first-order kinetic behavior. (e) Time-dependent change in the ratio of the integrated peaks areas of Ag(111) to Ag(200). (Reproduced with permission from ref. 39. Copyright 2016 American Chemical Society.)

In another example, Sun and co-workers recently reported a quantitative analysis of the nucleation and growth kinetics in the synthesis of Ag nanocrystals by integrating a microwave reactor with *in situ* HEXRD at a high-energy synchrotron beamline, as illustrated in Fig. 10a.^39^ This integrated technique could capture the kinetics of nanocrystal formation in large, statistically relevant solutions. The authors hypothesized that the peak areas measured from the HEXRD patterns were proportional to the mass of the corresponding crystalline materials in a pure phase. As a result, the time-dependent variation in peak area could be used to analyze the reaction kinetics occurring during nucleation and growth. Fig. 10b shows the HEXRD patterns of the resultant Ag nanocrystals recorded during the polyol reduction of AgNO_3_ at 140 °C at a number of key reaction time points. It can be seen that the diffraction signals ascribed to the (111), (200), and (220) planes of crystalline Ag started to appear at *t* = 11 min, implying that the nucleation process for the formation of stable seeds is very slow even at an elevated temperature. Fig. 10c shows the peak area of the Ag(111) reflection as a function of reaction time, respectively, which could be fitted well with a sigmoid function, except for *t* ≤ 15 min. Clearly, as shown in Fig. 10c, the formation of Ag nanocrystals involved four well-defined steps with specific kinetic features, that is, (i) induction of nucleation process, (ii) formation of seeds, (iii) growth of seeds, and (iv) completion of synthesis. In region-I (*t* = 0–10 min), no diffraction signals were detected, suggesting the formation of Ag nuclei as small as a few nanometers that tended to fluctuate at an elevated temperature. When some of the Ag nuclei grew large enough as to have an internal structure inert to thermal fluctuations, clear signals in the HEXRD patterns would appear. Such stable nuclei can also be referred to as seeds that were generated in region-II (*t* = 10–15 min). During this period, as shown in the insets of Fig. 10c, the trend for ln([Ag^+^]/[Ag^+^]_0_) with reaction time decreased linearly, suggesting that the polyol reduction of Ag^+^ ions followed the typical pseudo-first-order kinetic model. At *t* > 15 min, the linear relationship between ln([Ag^+^]/[Ag^+^]_0_) and reaction time no longer existed, revealing deviation from the pseudo-first-order kinetic model. Instead, a sigmoidal curve was observed, suggesting the occurrence of seed-mediated growth into larger nanocrystals with acceleration in reduction for the AgNO_3_ precursor through the autocatalytic route in region-III (*t* = 16–36 min) once the size of the Ag seeds had reached a critical value. Finally, in region-IV (*t* > 36 min), the growth rate slowed down to zero due to the full consumption of AgNO_3_ precursor, indicating the completion of the chemical reaction. The four well-distinguished steps were consistent with the analysis of the peak area for the Ag(200), as shown in Fig. 10d. It can be concluded that the nucleation and formation of seeds follows typical first-order reaction kinetics, while the autocatalytic kinetics dominate the behavior during the growth of seeds, which can be modelled using the Finke–Watzky kinetic theory,^42^ as discussed in Section 2.2.

Interestingly, by taking advantage of *in situ* HEXRD, the occurrence of anisotropic growth involved in the synthesis of Ag nanocrystals can be extrapolated from careful data analyses. In Fig. 10e, the ratio between the areas under the Ag(111) and Ag(200) peaks was plotted as a function of reaction time. The ratio showed a decrease from 2.6 to 1.8, and then at 18 min, a continuous increase to the steady-state value of 2.2 at 27 min. This data revealed that anisotropic growth varied with the reaction time. For example, the (111)/(200) ratios are larger than the equilibrium value during the stage of forming seeds (*i.e.*, region-II, *t* ≤ 15 min), suggesting that the surfaces of the Ag seeds were mainly terminated in {111} facets, which was also consistent with the TEM results. However, as recently reported by Mirsaidov and co-workers in the synthesis of Au and Ag nanocrystals, the homogeneous nucleation first gave rise to amorphous clusters, followed by their crystallization into seeds.^57^ This multistep nucleation process was directly observed in the solution by *in situ* TEM. In all, recording the crystalline diffraction signals using *in situ* HEXRD is interesting and it can reveal important aspects of the nanocrystal growth process such as increase in volume and change in shape anisotropy. However, since HEXRD cannot detect the possible intermediate solids with an amorphous structure, it cannot be applied to study the homogeneous nucleation process.

### 4.3. Transmission electron microscopy

In the third example, we discuss *in situ* liquid TEM, which is arguably the most promising method for directly observing and quantifying nanocrystal nucleation and growth. Thanks to the efforts from many groups, with the use of pulsed electrons triggered by an incident laser beam, imaging materials with speeds of several tens of milliseconds per frame has now become possible for liquid-phase syntheses.^57–61^ In 2009, Alivisatos and co-workers performed a classic experiment where the growth of a single Pt nanocrystal was tracked using *in situ* TEM in a liquid cell. They found that Pt nanocrystal grew either by monomer attachment or by coalescence between individual particles.^58^ Subsequently, Zheng and co-workers demonstrated real-time imaging of facet development for individual Pt nanocubes during growth by *in situ* TEM.^59^ In all, *in situ* TEM is currently one of the most commonly utilized characterization tools for obtaining the real-time structural evolution of nanocrystals.

In addition to real-time imaging, the electrons used during *in situ* TEM imaging can also be used as the reducing agent, where the electron flux (or dose) can serve an analogous role to that of the reducing agent concentration. This concept was demonstrated by Browning and co-workers in studying the synthesis of Ag nanocrystals by *in situ* TEM, where the electron beam was directly used to reduce aqueous AgNO_3_.^60^ The reduction mechanism can be explained through the radiochemical mechanism, as described in eqn (9):

9 e^–^ beam + H_2_O → e_aq_^–^, H_3_O^+^, H^–^, H_2_, OH^–^, H_2_O_2_

**Fig. 11:**
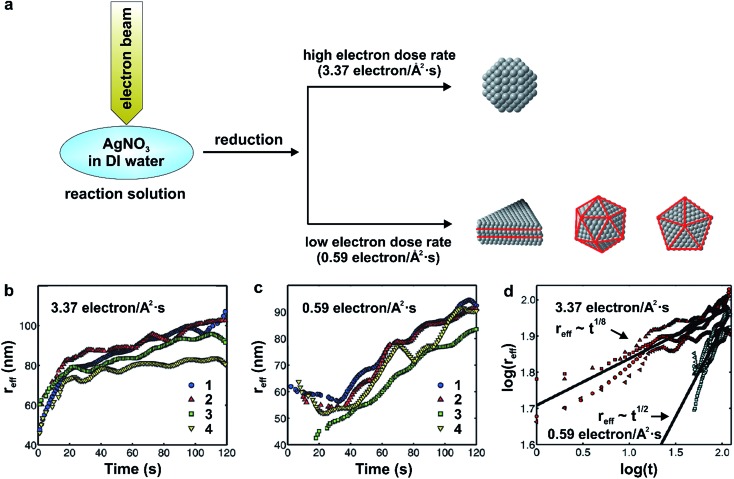
(a) Schematic illustration showing the dose-dependent formation of near-spherical Ag nanocrystals (high dose) and those with twinned structures including nanoplates, icosahedra, and pentagonal bipyramids (low dose). (b) Plot of the effective radius (*r*_eff_) as a function of time for four individual near-spherical Ag nanocrystals, with an electron dose rate at 3.37 electrons per Å^2^ per s. (c) Plot of the ‘*r*_eff_’ as a function of time (*t*) for four individual twinned Ag nanocrystals, with an electron dose rate at 0.59 electrons per Å^2^ per s. (d) Plot of the logarithmic relationships between the ‘*r*_eff_’ and ‘*t*’. The red data points correspond to panel (b), while the blue points correspond to panel (c). The black lines are the average power law fits for the four different nanocrystals (*r*_eff_ ∝ *t* ^1/8^ for the near-spherical nanocrystals and *r*_eff_ ∝ *t* ^1/2^ for the twinned nanocrystals) obtained by linear regression. (Reproduced with permission from ref. 60. Copyright 2012 American Chemical Society.)

The radiolysis of water by an electron beam (200 keV) forms free radicals and aqueous electrons (e_aq_^–^) that reduce the AgNO_3_ precursor to atoms, followed by their nucleation and then growth into nanocrystals. Similar to the role that the concentration of a reducing agent plays in a conventional synthesis, the dose rate of electron beam largely dictates the reduction rate and thus final shape of the nanocrystals. As shown in Fig. 11a, the high electron dose rate of 3.4 electrons per Å^2^ per s yielded the near-spherical nanocrystals while the low electron dose rate of 0.6 electrons per Å^2^ per s produced a mix of defect-lined nanocrystals, including nanoplates, icosahedra, and pentagonal bipyramids.

In addition, the direct visualization of growth dynamics acquired by *in situ* TEM allows the authors to quantitatively distinguish between reaction- and diffusion-limited growth of the near-spherical and defect-containing nanocrystals. Fig. 11b and c, trends the effective radius (*r*_eff_) as a function of reaction time at electron dose rates of 3.4 and 0.6 electrons per Å^2^ per s, respectively, for four individual nanocrystal growth trajectories. Importantly, as shown in Fig. 11d, when growth was conducted under the high beam current condition (*i.e.*, 3.4 electrons per Å^2^ per s), the effective radius of the near-spherical nanocrystals followed a *t* ^1/8^ power law, matching what is predicted using the Lifshitz–Slyozov–Wagner (LSW) theory for diffusion-limited growth, although the effective radius scaled as *t* ^1/8^, almost 3 times smaller than the *t* ^1/3^ scaling calculated for the purely diffusion-limited case in the LSW theory.^60,62^ In comparison, the effective radius for the defect-containing nanocrystals followed a power law as *t* ^1/2^ at a low electron dose rate of 0.6 electrons per Å^2^ per s, which is consistent with LSW theory for reaction-limited growth (*i.e.*, *r*_eff_ ∝ *t* ^1/2^). In this case of sluggish reduction, it should be concluded that the Ag^+^ ions were able to seek the preferred nucleation sites as the reduction was the rate limiting step (the Ag^+^ ions did not react instantly at the nanocrystal surface), and tend to induce hcp packing to generate nanocrystals with internal defects, which is similar to our results in the polyol synthesis of Pd nanocrystals,^23^ as discussed in Section 3.1.

Although major achievements have been made by directly observing the nucleation and growth of nanocrystals using *in situ* TEM,^63^ great caution must be practiced when establishing generalized conclusions, as some artifacts may be introduced by the electron beam.^64,65^ The foremost concern is radiation damage incurred by the specimen (*e.g.*, solvent, precursors, and nanocrystals),^61^ which may profoundly impact the outcome of an experiment. For example, the rates of *in situ* galvanic replacement between Pd-ions and Ag nanoparticles were much higher compared to *ex situ* control experiments, which indicates that the electron beam strongly affects galvanic-type processes in the liquid cell.^66^ In addition, due to the small size of the holder, it is also possible for the nanostructures to interact with or even attach to the walls of the TEM-specimen chamber, blocking reagents from a portion of the growing nanocrystals and potentially resulting in asymmetric growth patterns and non-uniform products. A good practice may be to carry out parallel *in situ* and *ex situ* experiments under the same conditions in an effort to sort out the additional affects introduced by the electron beam and TEM specimen holder.

Although much effort has been devoted to the investigation of the nucleation and growth process using *in situ* characterization tools, as mentioned above, these *in situ* tools used individually cannot achieve a comprehensive understanding of the formation mechanisms of nanocrystals. For example, XAFS is inadequate to provide the information regarding the size and shape evolution of nanocrystals. In general, these tools should be used in conjunction to gather a more complete picture for what is happening inside a reaction mixture. In all, it is safe to say that in order to acquire adequate information regarding the size, shape, structure, composition, and all the temporal intermediates formed during the nucleation and growth of nanocrystals, we must work towards the development of highly integrated *in situ* tools, that can extract all the relevant parameters in real-time to capture the dynamic aspects and true nature of nanocrystal nucleation and growth within the reaction solution.

## 5. Summary and outlook

In this *Perspective* article, we have discussed how colloidal metal nanocrystals with specific features can be deterministically synthesized through a *quantitative* analysis of the reduction kinetics. At the beginning of this article, we discuss how ICP-MS and UV-vis measurements can be used to quantify the reduction kinetics of the precursor in a synthesis, together with some of the potential problems involved in such measurements, as well as some possible solutions. We then demonstrate how the kinetic parameters can be derived through curve fitting. Subsequently, we strive to establish the relationships between the reduction rate of the precursor and the structural features of the resultant nanocrystals, including one-pot and seed-mediated syntheses involving one-shot or dropwise addition of the precursor. Most importantly, through case studies, we were able to showcase the true power of using the reduction rate of precursor as a quantitative knob to controllably manipulate the shape and structure of metal nanocrystals. Furthermore, we also present a number of examples to highlight the use of *in situ* characterization tools for quantitatively studying the nucleation and growth dynamics in real time.

Although significant progress has been made towards correlating key experimental parameters with the nanocrystal products, a number of scientific problems or technological challenges still need to be addressed before we are able to use quantitative methods for all the different synthetic systems. One of the major challenges is how to determine the reduction kinetics of high-valent precursor compounds (such as PtCl_6_^2–^ that is widely used in the synthesis of Pt-based nanocatalysts) through spectroscopic measurements.^67,68^ Data acquisition and analyses in such cases, for example, are difficult due to the possible coexistence of PtCl_6_^2–^ and PtCl_4_^2–^ ions and their individual interaction with the nuclei or seeds during the transformation from PtCl_6_^2–^ to PtCl_4_^2–^, and then finally to Pt atoms that are responsible for the nucleation and growth of nanocrystals. This multi-step evolution pathway is very difficult to model due to the high complexity.^67^ A possible solution to help determine the reduction kinetics of such high-valent precursors is to simultaneously use ICP-MS and UV-vis measurements while developing more complicated mathematical models.

Another challenge is precursor hydrolysis and thus formation of hydrated species during the synthesis may often occur when the solvent is water. For example, the chloride ions around the Pt- or Pd-ions in precursors such as PtCl_6_^2–^, PtCl_4_^2–^, or PdCl_4_^2–^ can be partially or completely replaced by H_2_O, which is expected to have a pronounced impact on the reduction kinetics of the precursor, and subsequently, the outcome of an aqueous-phase synthesis.^43–45,67,69^ A detailed study on the effect of hydrolysis should be seriously considered as it will provide useful information to better understand the reduction mechanism of the salt precursor. When these challenges are fully addressed, it will become possible to rationally design, synthesize, and engineer nanocrystals with desired and expected properties by dialing in the exact experimental parameters. Ultimately, we hope that our perspective, together with the case studies and concepts presented herein, will provide excitement and inspiration for future work that will push the field of nanocrystal synthesis away from the trial-and-error approach and towards a deterministic research with quantitative measure and prediction power.

## Conflicts of interest

There are no conflicts to declare.

## References

[cit1] Ahmadi T. S., Wang Z. L., Green T. C., Henglein A., El-Sayed M. A. (1996). Science.

[cit2] Sun Y., Xia Y. (2002). Science.

[cit3] Zhang L., Roling L. T., Wang X., Vara M., Chi M., Liu J., Choi S. I., Park J., Herron J. A., Xie Z., Mavrikakis M., Xia Y. (2015). Science.

[cit4] Yavuz M. S., Cheng Y., Chen J., Cobley C. M., Zhang Q., Rycenga M., Xie J., Kim C., Song K. H., Schwartz A. G., Wang L. V., Xia Y. (2009). Nat. Mater..

[cit5] Xia Y., Xiong Y., Lim B., Skrabalak S. E. (2009). Angew. Chem., Int. Ed..

[cit6] Xia Y., Xia X., Peng H. C. (2015). J. Am. Chem. Soc..

[cit7] Liu P., Qin R., Fu G., Zheng N. (2017). J. Am. Chem. Soc..

[cit8] Gilroy K. D., Ruditskiy A., Peng H. C., Qin D., Xia Y. (2016). Chem. Rev..

[cit9] Xia Y., Gilroy K. D., Peng H. C., Xia X. (2017). Angew. Chem., Int. Ed..

[cit10] Stamenkovic V. R., Fowler B., Mun B. S., Wang G., Ross P. N., Lucas C. A., Markovic N. M. (2007). Science.

[cit11] Crespo-Quesada M., Yarulin A., Jin M., Xia Y., Kiwi-Minsker L. (2011). J. Am. Chem. Soc..

[cit12] Gilroy K. D., Peng H. C., Yang X., Ruditskiy A., Xia Y. (2017). Chem. Commun..

[cit13] Ruditskiy A., Peng H. C., Xia Y. (2016). Annu. Rev. Chem. Biomol. Eng..

[cit14] Quan Z., Wang Y., Fang J. (2013). Acc. Chem. Res..

[cit15] Zhang H., Jin M., Xia Y. (2012). Angew. Chem., Int. Ed..

[cit16] Lamer V. K., Dinegar R. H. (1950). J. Am. Chem. Soc..

[cit17] Lee J., Yang J., Kwon S. G., Hyeon T. (2016). Nat. Rev. Mater..

[cit18] Liu M., Guyot-Sionnest P. (2005). J. Phys. Chem. B.

[cit19] Ming T., Feng W., Tang Q., Wang F., Sun L., Wang J., Yan C. (2009). J. Am. Chem. Soc..

[cit20] Langille M. R., Personick M. L., Zhang J., Mirkin C. A. (2012). J. Am. Chem. Soc..

[cit21] Xia X., Choi S. I., Herron J. A., Lu N., Scaranto J., Peng H. C., Wang J., Mavrikakis M., Kim M. J., Xia Y. (2013). J. Am. Chem. Soc..

[cit22] Huang H., Wang Y., Ruditskiy A., Peng H. C., Zhao X., Zhang L., Liu J., Ye Z., Xia Y. (2014). ACS Nano.

[cit23] Wang Y., Peng H. C., Liu J., Huang C. Z., Xia Y. (2015). Nano Lett..

[cit24] Ruditskiy A., Zhao M., Gilroy K. D., Vara M., Xia Y. (2016). Chem. Mater..

[cit25] Hsu S. C., Chuang Y. C., Sneed B. T., Cullen D. A., Chiu T. W., Kuo C. H. (2016). Nano Lett..

[cit26] Timoshkin A. Y., Kudrev A. G. (2012). Russ. J. Inorg. Chem..

[cit27] Vara M., Lu P., Yang X., Lee C. T., Xia Y. (2017). Chem. Mater..

[cit28] Wiley B., Sun Y., Xia Y. (2005). Langmuir.

[cit29] Wiley B., Herricks T., Sun Y., Xia Y. (2004). Nano Lett..

[cit30] Zhou M., Wang H., Vara M., Hood Z. D., Luo M., Yang T. H., Bao S., Chi M., Xiao P., Zhang Y., Xia Y. (2016). J. Am. Chem. Soc..

[cit31] Zhu C., Zeng J., Tao J., Johnson M. C., Schmidt-Krey I., Blubaugh L., Zhu Y., Gu Z., Xia Y. (2012). J. Am. Chem. Soc..

[cit32] Zeng J., Zhu C., Tao J., Jin M., Zhang H., Li Z. Y., Zhu Y., Xia Y. (2012). Angew. Chem., Int. Ed..

[cit33] Peng H. C., Park J., Zhang L., Xia Y. (2015). J. Am. Chem. Soc..

[cit34] Lv T., Yang X., Zheng Y., Huang H., Zhang L., Tao J., Pan L., Xia Y. (2016). J. Phys. Chem. C.

[cit35] Xie S., Peng H. C., Lu N., Wang J., Kim M. J., Xie Z., Xia Y. (2013). J. Am. Chem. Soc..

[cit36] Pacławski K., Sak T. (2015). J. Min. Metall., Sect. B.

[cit37] Boruah S. K., Boruah P. K., Sarma P., Medhi C., Medhi O. K. (2012). Adv. Mater. Lett..

[cit38] Harada M., Kizaki S. (2016). Cryst. Growth Des..

[cit39] Liu Q., Gao M. R., Liu Y., Okasinski J. S., Ren Y., Sun Y. (2016). Nano Lett..

[cit40] H Yang T., Peng H. C., Zhou S., Lee C. T., Bao S., Lee Y. H., Wu J. M., Xia Y. (2017). Nano Lett..

[cit41] Ozkar S., Finke R. G. (2016). Langmuir.

[cit42] Watzky M. A., Finke R. G. (1997). J. Am. Chem. Soc..

[cit43] Elding L. I., Olsson L. F. (1978). J. Phys. Chem. A.

[cit44] Kriek R. J., Mahlamvana F. (2012). Appl. Catal., A.

[cit45] le Roux C. J., Kriek R. J. (2017). Hydrometallurgy.

[cit46] Baletoo F., Ferrando R. (2005). Rev. Mod. Phys..

[cit47] Xia X., Xie S., Liu M., Peng H. C., Lu N., Wang J., Kim M. J., Xia Y. (2013). Proc. Natl. Acad. Sci. U. S. A..

[cit48] Baletto F., Ferrando R., Fortunelli A., Montalenti F., Mottet C. (2002). J. Chem. Phys..

[cit49] Gilroy K. D., Elnabawy A. O., Yang T. H., Roling L. T., Howe J., Mavrikakis M., Xia Y. (2017). Nano Lett..

[cit50] Niu Z., Peng Q., Gong M., Rong H., Li Y. (2011). Angew. Chem., Int. Ed..

[cit51] Peng H. C., Xie S., Park J., Xia X., Xia Y. (2013). J. Am. Chem. Soc..

[cit52] Ortiz N., Weiner R. G., Skrabalak S. E. (2014). ACS Nano.

[cit53] Kim D., Resasco J., Yu Y., Asiri A. M., Yang P. (2014). Nat. Commun..

[cit54] Yao T., Liu S., Sun Z., Li Y., He S., Cheng H., Xie Y., Liu Q., Jiang Y., Wu Z. (2012). J. Am. Chem. Soc..

[cit55] Yao T., Sun Z., Li Y., Pan Z., Wei H., Xie Y., Nomura M., Niwa Y., Yan W., Wu Z. (2010). J. Am. Chem. Soc..

[cit56] Marbella L. E., Chevrier D. M., Tancini P. D., Shobayo O., Smith A. M., Johnston K. A., Andolina C. M., Zhang P., Mpourmpakis G., Millstone J. E. (2015). J. Am. Chem. Soc..

[cit57] Lou N. D., Sen S., Bosman M., Tan S. F., Zhong J., Nijhuis C. A., Krai P., Matsudaira P., Mirsaidov U. (2017). Nat. Chem..

[cit58] Zheng H., Smith R. K., Jun Y. W., Kisielowski C., Dahmen U., Alivisatos A. P. (2009). Science.

[cit59] Liao H. G., Zherebetskyy D., Xin H. L., Czarnik C., Ercius P., Elmlund H., Pan M., Wang L. W., Zheng H. M. (2014). Science.

[cit60] Woehl T. J., Evans J. E., Arslan I., Ristenpart W. D., Browning N. D. (2012). ACS Nano.

[cit61] Jonge N. D., Ross F. M. (2011). Nat. Nanotechnol..

[cit62] Baldan A. (2002). J. Mater. Sci..

[cit63] Ross F. M. (2015). Science.

[cit64] Woehl T. J., Jungjohann K. L., Evans J. E., Arslan I., Ristenpart W. D., Browning N. D. (2013). Ultramicroscopy.

[cit65] Noh K. W., Liu Y., Sun L., Dillon S. J. (2012). Ultramicroscopy.

[cit66] Sutter E., Jungjohann K., Bliznakov S., Courty A., Maisonhaute E., Tenney S., Sutter P. (2014). Nat. Commun..

[cit67] Chen S., Yang Q., Wang H., Zhang S., Li J., Wang Y., Chu W., Ye Q., Song L. (2015). Nano Lett..

[cit68] Xie S., Choi S. I., Lu N., Roling L. T., Herron J. A., Zhang L., Park J., Wang J., Kim M. J., Xie Z., Mavrikakis M., Xia Y. (2014). Nano Lett..

[cit69] Mahlamvana F., Kriek R. J. (2014). Appl. Catal., B.

